# The Class II Trehalose 6-phosphate Synthase Gene *PvTPS9* Modulates Trehalose Metabolism in *Phaseolus vulgaris* Nodules

**DOI:** 10.3389/fpls.2016.01589

**Published:** 2016-11-01

**Authors:** Aarón Barraza, Cecilia Contreras-Cubas, Georgina Estrada-Navarrete, José L. Reyes, Marco A. Juárez-Verdayes, Nelson Avonce, Carmen Quinto, Claudia Díaz-Camino, Federico Sanchez

**Affiliations:** ^1^Departamento de Biología Molecular de Plantas, Instituto de Biotecnología/Universidad Nacional Autónoma de MéxicoCuernavaca, Mexico; ^2^Centro de Investigación en Dinámica Celular, Universidad Autónoma del Estado de MorelosCuernavaca, Mexico

**Keywords:** Class II trehalose-6-phosphate synthase, trehalose biosynthetic pathway, legume-rhizobium symbiosis signaling, AGO1-complex, *Phaseolus vulgaris* (common bean)

## Abstract

Legumes form symbioses with rhizobia, producing nitrogen-fixing nodules on the roots of the plant host. The network of plant signaling pathways affecting carbon metabolism may determine the final number of nodules. The trehalose biosynthetic pathway regulates carbon metabolism and plays a fundamental role in plant growth and development, as well as in plant-microbe interactions. The expression of genes for trehalose synthesis during nodule development suggests that this metabolite may play a role in legume-rhizobia symbiosis. In this work, *PvTPS9*, which encodes a Class II trehalose-6-phosphate synthase (TPS) of common bean (*Phaseolus vulgaris*), was silenced by RNA interference in transgenic nodules. The silencing of *PvTPS9* in root nodules resulted in a reduction of 85% (± 1%) of its transcript, which correlated with a 30% decrease in trehalose contents of transgenic nodules and in untransformed leaves. Composite transgenic plants with *PvTPS9* silenced in the roots showed no changes in nodule number and nitrogen fixation, but a severe reduction in plant biomass and altered transcript profiles of all Class II *TPS* genes. Our data suggest that *PvTPS9* plays a key role in modulating trehalose metabolism in the symbiotic nodule and, therefore, in the whole plant.

## Introduction

Legumes have symbiotic relationships with rhizobial soil bacteria, which results in the formation of nitrogen-fixing nodules on the roots of the plant host. Thanks to this remarkable association, leguminous plants can grow in nitrogen-poor environments. However, the biological fixation of nitrogen is energetically expensive; to support growth, plants must maintain a delicate balance that ensures sufficient fixed nitrogen without depleting their carbon reserves. One way in which legumes achieve this balance is by regulating the final number of nodules in the root (Gage, 2004; Ferguson et al., 2010; Kouchi et al., 2010).

Molecular genetic analyses in model legume species have shown that legumes inhibit nodulation by systemic signals. In the autoregulation of nodulation feedback, a signal produced in incipient root nodules induces the production of an inhibitory signal in the shoot; this signal is transported to the root and limits subsequent rhizobial infection and nodulation (Limpens and Bisseling, 2003; Searle et al., 2003; Ferguson et al., 2010). The extent of nodulation is also regulated by the nitrate content in the soil (Gibson and Harper, 1985; Imsande, 1986; Streeter, 1988). Thus, disparities in the carbon/nitrogen balance or a decline in the nitrogen demand may result in decreased photosynthate supply from shoot to nodules, arresting their growth and development (Bacanamwo and Harper, 1996, 1997).

The trehalose biosynthetic pathway functions as a major regulator of carbon metabolism and thus plays a fundamental role in plant growth and development (Wahl et al., 2013; Lunn et al., 2014; Figueroa et al., 2016; Figueroa and Lunn, 2016). Trehalose, a non-reducing glucose disaccharide, occurs widely in nature. The most common route for trehalose biosynthesis involves transfer of UDP-glucose to glucose-6-phosphate by the enzyme trehalose-6-phosphate synthase (TPS) to form trehalose-6-phosphate. Dephosphorylation of trehalose-6-phosphate by trehalose-6-phosphate phosphatase (TPP) yields free trehalose. In plants, multi-gene families encode TPS and TPP (Leyman et al., 2001). *TPS* genes include Class I genes, which usually encode catalytically active TPS enzymes, and Class II genes, which encode proteins that do not possess TPS or TPP enzymatic activity but contain TPS and TPP domains (Avonce et al., 2010). The Class III family encodes functional TPP enzymes (Vogel et al., 1998; Vandesteene et al., 2012). The catabolism of trehalose is mediated by the enzyme trehalase (Van Houtte et al., 2013).

Interestingly, various microorganisms, including rhizobia, produce trehalose during their symbiotic or pathogenic interactions with plants (Brodmann et al., 2002; Foster et al., 2003; Ocón et al., 2007; Wilson et al., 2007, 2010; Nehls, 2008; Barraza et al., 2013; Garg and Singla, 2016). Free-living rhizobia under physiological stress produce high levels of trehalose and this metabolite plays an important role as osmoprotectant in the development of symbiotic nitrogen-fixing root nodules (Sugawara et al., 2010) or during nodule senescence (Müller et al., 2001). Although, most of the trehalose in nitrogen-fixing nodules is produced by the symbiotic bacteria (Suárez et al., 2008; López et al., 2009), increases of diverse trehalose-related transcripts have been reported in other tissues of legume plants living in symbiosis with rhizobia (Pontius et al., 2003; Ramírez et al., 2005; Hernández et al., 2007; Thibivilliers et al., 2009; O'Rourke et al., 2014).

Previously, we studied the effect of modifying the endogenous expression levels of trehalase (*PvTRE1*) in composite common bean plants. Diminishing the transcript levels of *PvTRE1* in transgenic nodules results in increased trehalose contents and substantially improves the nitrogen-fixation rate without affecting plant performance (Barraza et al., 2013). In this work, we silenced the expression of a Class II *TPS* gene (*PvTPS9*) in nodules of common bean elicited by *Rhizobium etli* strain CFN42, and observed important systemic modifications in the expression of all Class II *TPS* genes as well as in the final contents of trehalose. Interestingly, although neither nodule number nor nitrogen fixation rates were altered, plant biomass was severely reduced. Altogether, the deleterious effects observed in composite common bean plants caused by reducing the expression of *PvTPS9* in root nodules suggest that *PvTPS9* plays an important role in modulating trehalose metabolism in the symbiotic nodule and, therefore, throughout the plant.

## Materials and methods

### Plant materials and growth conditions

*Phaseolus vulgaris* cv. Negro Jamapa (common bean) composite plants were used in this study. The generation of composite common bean plants was achieved following the protocol published by Estrada-Navarrete et al. (2007). Common bean seeds were surface disinfected and germinated under sterile conditions for 2 days and then transferred to pots with vermiculite. Plantlets were grown in a greenhouse under natural photoperiod and 26–28°C temperature, and watered with Broughton & Dilworth (B&D) nutrient solution (Broughton and Dilworth, 1971). Hairy roots of 3- to 6-cm emerging from the cotyledonary node of common bean seedlings infected with *Agrobacterium rhizogenes* strain K599 were obtained after 2 weeks. Transgenic roots were identified by epifluorescence microscopy following the signal emitted by the reporter gene [encoding Tomato Fluorescent Protein (TdT), Valdés-López et al., 2008]. Wild-type and untransformed roots were excised and the composite plants were planted in new pots containing fresh vermiculite. Immediately afterwards, transformed roots were directly inoculated by adding 1 mL of 5 × 10^8^ mL^−1^ *R. etli* strain CFN42 culture, and grown for 21 d. Composite bean plants were regularly watered with a B&D nitrogen-free nutrient solution (without the addition of KNO_3_). Transgenic roots and nodules were collected in liquid nitrogen, finely ground with a mortar and pestle and stored at −80°C until they were used for quantitative RT-PCR (qRT-PCR) and carbohydrate profile analysis by high-performance liquid chromatography (HPLC). Alternatively, transgenic nodules were collected, formaldehyde-fixed, and embedded in LR-White for detailed characterization by optical and confocal microscopy, and also by transmission electron microscopy (TEM).

### *In silico* identification of the Trehalose-6-Phosphate Synthase gene families in common bean and phylogenetic analysis

We identified expressed sequence tags (ESTs) corresponding to a *Trehalose-6-Phosphate Synthase* (*TPS*) gene expressed in common bean nodules (Ramírez et al., 2005; Hernández et al., 2007), which is presumably involved in the biosynthesis of trehalose, using the common bean Gene Index v.4.0 (DFCI, http://compbio.dfci.harvard.edu/tgi/). In addition, by analyzing the whole common bean genome (Phytozome v11, http://www.phytozome.net; Mazorka, http://mazorka.langebio.cinvestav.mx/blast/), we identified the complete coding sequence of seven *TPS*-paralogous genes. The coding sequences of these putative *TPS* genes were analyzed with Pfam (http://pfam.xfam.org), and then analyzed by BLAST with other TPS sequences deposited in GenBank using the BLASTX tool at the National Center for Biotechnology Information (NCBI, https://blast.ncbi.nlm.nih.gov) website. Multiple sequence alignment of the full-length TPS protein sequences was performed using ClustalX 1.83 (Thompson et al., 1997) and manually edited using Seaview (Galtier et al., 1996).

Phylogenetic trees were generated using MrBayes 3 (Ronquist and Huelsenbeck, 2003) and PhyML 3.0 (Guindon et al., 2010). In the first run of MrBayes3, all available fixed-rate amino acid substitution models were explored, clearly showing the Whelan and Goldman model produced the best fit for the data. In a second iteration, the program was executed in two independent runs with six chains for 7500 generations using the Jones-Taylor-Thornton model. Although, the phylogenetic trees were sampled every 100 generations, the first 500 were discarded. The remaining 7000 were used to build a consensus phylogenetic tree that allowed calculation of the posterior probability of all bipartitions. PhyML 3.0 is designed to calculate maximum likelihood phylogenies for large data sets using a Hill-Climbing algorithm. This program was run with 100 bootstraps and the Jones-Taylor-Thornton substitution model was used.

### Molecular chimeras and plant transformation

The 3′-untranslated region (3′-UTR) of *PvTPS9* (300 bp, Figure S1) was amplified by PCR from complementary DNA (cDNA) synthesized from total RNA obtained from 21 days post-inoculation (dpi)- root nodules elicited with *R. etli* strain CFN42. DNA amplification was achieved using a combination of *Pv*TPS9RiFwd and *Pv*TPS9RiRev primers (Table S1, Figure S1). The resulting 300-bp DNA fragment was cloned into pENTR/SD/D-TOPO (Invitrogen, Carlsbad, CA, U.S.A.) and later recombined into the pTdT-DC-RNAi binary vector (Valdés-López et al., 2008). The orientation of the inserted 3′UTR of *PvTPS9* in this vector was confirmed by PCR using the WRKY-5-Rev and *Pv*TPS9RiFwd primers (Table S1). The open reading frame of the *otsA* gene was obtained by amplification with PCR using genomic DNA from *R. etli* as template and a combination of *Re*TPSFor and *Re*TPSRev primers (Table S1). The resulting 1.4 Kb DNA fragment was cloned into pCAMBIA-1304 vector to produced the p35S::*Re*TPS molecular construct. The procedure to generate the *Pv*TRE1-RNAi plasmid has been described by Barraza et al. (2013). Plasmids were introduced by electroporation into *A. rhizogenes* strain K599, and used for plant transformation as described.

### Bacterial strains and cultures

*R. etli* strain CFN42 was grown in peptone-yeast extract (PY) liquid culture [0.5% bactopeptone (w/v), 0.3% yeast extract (w/v), 7 mM CaCl_2_·2H_2_O] supplemented with 20 μg/mL nalidixic acid at 30°C to a cell density of 5 × 10^8^ mL^−1^. *A. rhizogenes* K599 was grown in LB solid culture at 30°C for 24 h. *A. rhizogenes* K599 transformed with pTdT-DC-RNAi, *A. rhizogenes* K599 with pTdT-GUS-RNAi, and *A. rhizogenes* K599 with pTdT-*PvTPS9*-RNAi were grown in LB solid culture supplemented with 200 μg/mL spectinomycin at 30°C for 24 h. All these *A. rhizogenes* K599 strains were used to generate composite common bean plants.

### *In silico* identification of *Pv*miR172 in common bean

The microRNA172 (miRNA172) was previously identified in leguminous plants (Lu and Yang, 2010; Peláez et al., 2012). Its presence in common bean was confirmed by analyzing the whole *Phaseolus vulgaris* genome (Phytozome v11, http://www.phytozome.net; Mazorka, http://mazorka.langebio.cinvestav.mx/blast/), and the retrieved sequence of *Pv*miR172 was later analyzed in miRBase (http://www.mirbase.org/search.shtml).

### TEM and optical microscopy

Nodules at 21 dpi (9 nodules per experiment collected from different composite bean plants) were embedded in LR-White resin after being fixed with 2% *p*-formaldehyde and 0.4% glutaraldehyde in PBS buffer for 2 h at room temperature, and subjected to dehydration in a graded ethanol series. For optical microscopy, tissue sections of 7 μm were stained with toluidine blue. The optical microscopy analyses were performed with a light microscope (Motic BA300, Xiamen, China) and photographed with a digital camera (Motic M1000, Xiamen, China). Samples for transmission electron microscopy (TEM) were stained with uranyl acetate. Thin sections of 60 nm were prepared with an ultramicrotome (Leica Ultracut R) and stained with uranyl acetate. TEM analyses were performed with a Zeiss EM900 transmission electron microscope dual vision coupled cam system (Gatan, Inc., Pleasanton, CA, U.S.A.).

### Culture of rhizobium re-isolated from transgenic nodules and determination of colony forming units

Nodules at 21 dpi (9 nodules per experiment collected from different composite bean plants) were surface-disinfected by immersion in sodium-hypochlorite (10% v/v) for 10 min. Then, each nodule was homogenized in 5 volumes of 100 mM MgCl_2_ using a plastic pipette. For each nodule, 100 μL of a serial dilution (10^0^–10^−8^) were plated onto PY solid medium supplemented with 20 μg/mL nalidixic acid and incubated for 24 h at 30°C. Later, *R. etli* CFN42 colony-forming units were determined.

### Analysis of the carbohydrate profile of composite common bean plants by high-performance liquid chromatography

Nodules and leaves from composite common bean plants harvested 21 dpi were collected, weighed, frozen in liquid nitrogen, and finely ground. Samples were mixed with 1 mL 80% ethanol (v/v) for 10 min at 80°C. The supernatant was dried, dissolved in 1 mL HPLC grade water, and filtered through a 0.22-μm membrane to remove impurities. These extracts were analyzed by high-performance liquid chromatography (HPLC) in a Waters-600E system controller (Waters, Milford, MA, U.S.A.) equipped with a Waters 410 refractive index detector (Waters, Milford, MA, U.S.A.) and a carbohydrate analysis column (Kromasil NH2–5 mm, Supelco, St. Louis, MO, U.S.A.). The temperature of the column was kept at 35°C during the analysis. The mobile phase used in these assays was acetonitrile:water (80:20) at a flow rate of 1.2 mL/min. Glucose, fructose, sucrose, maltose, and trehalose (Sigma-Aldrich) standard solutions were used as reference points. Standard curves used to carry out the quantifications had a correlation index ranging from 0.987 to 0.993. Results were expressed as μg/mg of fresh weight.

### Efficiency of symbiotic N_2_ fixation of transformed roots

Nitrogen fixation was assayed using the acetylene reduction method (Vessey, 1994). Briefly, 21 dpi transgenic roots inoculated with *R. etli* CFN42 (9 individual roots per experiment) were placed in a 160-mL vial closed with a serum cap. Four milliliters of air was withdrawn from the closed vial and replaced by acetylene gas. Ethylene production was assayed in a gas chromatograph and expressed as nanomoles of ethylene per minute per nodule of fresh weight.

### Quantitative PCR assays

RNA from plant tissues was extracted using TRIzol (Invitrogen, Carlsbad, CA, U.S.A.). RNA quantity was measured spectrophotometrically, and only RNA samples with a 260/280 ratio of between 1.9 and 2.1 and a 260/230 ratio of greater than 2.0 were used. The integrity of RNA samples was confirmed by agarose gel electrophoresis. For reverse transcription, total RNA was treated with DNaseI (Invitrogen), and transcribed using the Revert Aid H Minus First-strand cDNA synthesis kit (Fermentas) with anchored-oligo (dT)_18_ primer, according to the manufacturer's instructions. For qRT-PCR analysis, a LightCycler480 Real-time PCR system (Roche, Penzberg, Germany) was used. qRT-PCR was performed according to the Maxima SYBR Green qPCR Master Mix (2X) protocol (Thermo Scientific, Waltham, MA, U.S.A.). A control sample with no DNA was used in each assay. Primers are listed in Table S1. In general, qPCR data came from six independent (*n* = 6) experimental replicas. The statistical significance was determined with an unpaired two-tailed Student's *t-*test. Each biological replicate was tested in triplicate and data were normalized to the *Elongation factor 1-alpha* (*PvEF1a*) reference gene (Livak and Schmittgen, 2001).

### Common bean argonaute (*Pv*AGO1) immunoprecipitation followed by qRT-PCR analysis

The immunoprecipitation (IP) assays were carried out according to Qi and Mi (2010) with some minor modifications (Contreras-Cubas et al., 2012). Briefly, 80 mg ground tissue from 14 and 22 dpi nodules was homogenized with 1 mL extraction buffer [50 mM Tris-HCl pH 7.5, 1.5 mM NaCl, 0.1% Nonidet 40, 4 mM MgCl_2_, 5 mM DTT, 1 tablet/10 mL protease inhibitor (Roche, Penzberg Germany)]. The homogenate was centrifuged to 19,083 × g for 25 min to pellet the cellular debris. The supernatant was incubated with 5 μL protein A-agarose pearls (Roche, Penzberg Germany) for 1 h at 4°C with constant rotation. Then, this mixture was centrifuged at 250 × g for 5 min to remove nonspecific Protein A-binding proteins. A 1:50 final dilution of anti-*Pv*AGO1 antibody with 10 μL protein was added to the supernatant and incubated overnight at 4°C with constant rotation. Material bound to the Protein A-agarose pearls was washed with 1 mL extraction buffer three times. Protein complexes bound to the A-agarose pearls (IP samples) were resuspended in 30 μL extraction buffer and treated with TRIzol (Invitrogen, Carlsbad, CA, U.S.A.) for RNA extraction.

The RNA purified from the IP samples was used for cDNA synthesis with the Ncode miRNA First-strand cDNA synthesis kit (Invitrogen, Carlsbad, CA, U.S.A.) according to the manufacturer's recommendations. The Ncode kit is designed to detect the mature form of the miRNA of interest, but not the precursor molecule. To remove DNA, the RNA was treated with DNaseI as indicated (Invitrogen, Carlsbad, CA, U.S.A.). The absence of DNA in these samples was confirmed by a simple PCR assay (preheating for 5 min at 95°C, followed by 40 cycles of denaturing for 15 s at 95°C, annealing and elongation for 15 s at 55.8°C) using Taq-DNA polymerase (Fermentas Life Sciences, Waltham, MA, U.S.A). cDNA synthesis was performed using the standard First Strand cDNA Synthesis kit (Fermentas) according to the manufacturer's protocol. For qRT-PCR analysis, a LightCycler480 Real-time PCR system (Roche, Penzberg, Germany) was used. The qRT-PCR was performed according to the Maxima SYBR Green qPCR Master Mix (2X) protocol (Thermo Scientific, Waltham, MA, U.S.A.). A control sample with no DNA was used in each assay. The primers are listed in Table S1. The miRNAs co-immunoprecipitated with the anti-*Pv*AGO1 antibody were derived from three independent (*n* = 3) biological replicates. Each biological replicate was tested in triplicate technical replicates and data were normalized to *Pv*miR2118 and *Pv*miR172 (Arenas-Huertero et al., 2009). The statistical significance of the obtained results was determined with an unpaired two-tailed Student's *t*-test.

## Results

### Identifying plant trehalose biosynthetic genes in nitrogen-fixing nodules of common bean

**Figure 1:**
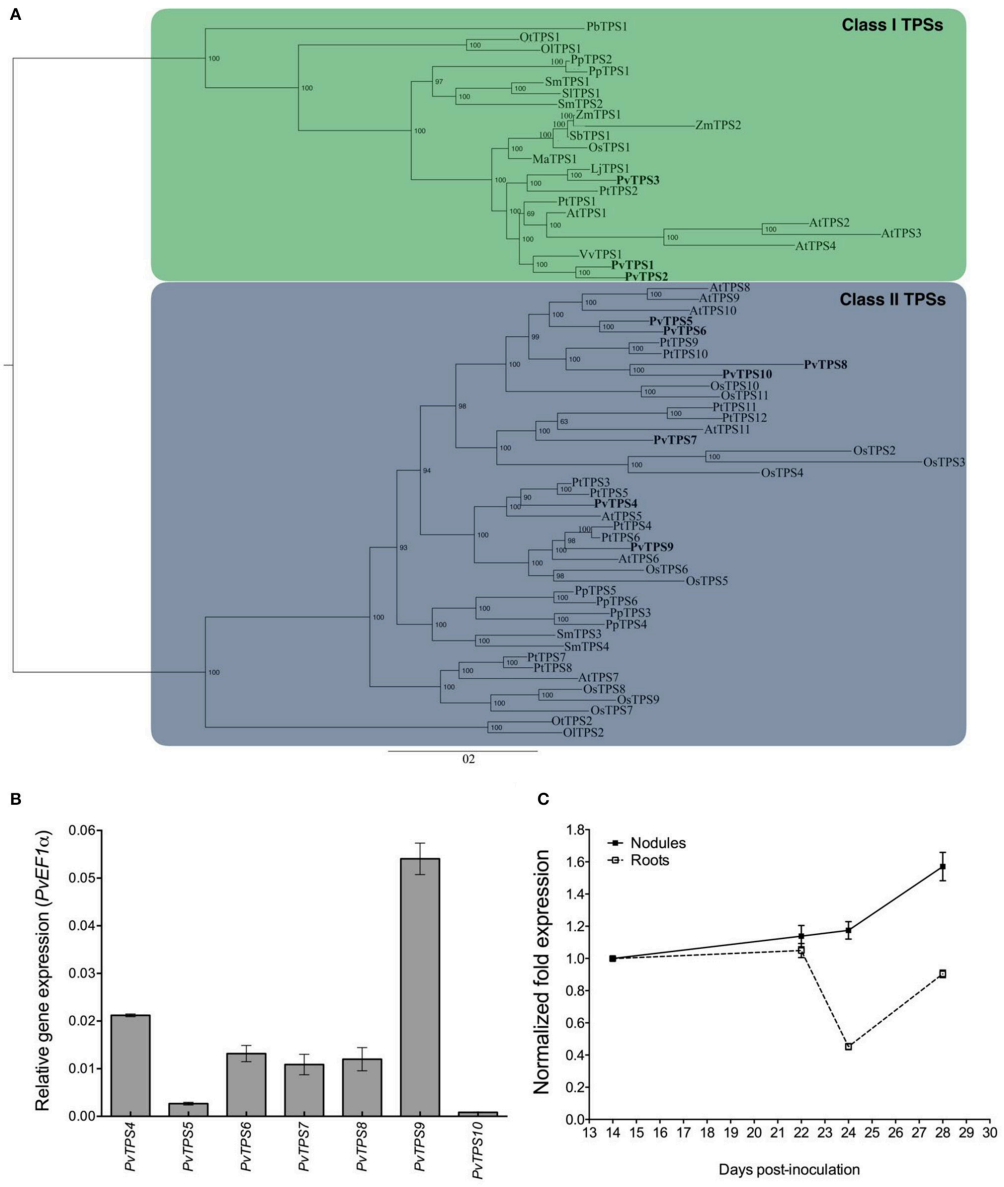
***Phaseolus vulgaris* Class II TPS9 (*Pv*TPS9) phylogeny and gene expression during nodulation. (A)** *Pv*TPS9 phylogenetic analysis. Multiple sequence alignment of all full-length TPS protein sequences was performed using ClustalX 1.83 (Thompson et al., 1997) and manually edited using Seaview (Galtier et al., 1996). *Pv*TPS9 phylogenetic analysis was conducted using MrBayes 3 and PhyML 3.0 (Ronquist and Huelsenbeck, 2003; Guindon et al., 2010, respectively), as described in Materials and Methods. The scale on the x-axis represents the estimated branch lengths and numbers indicate the bootstrap values. The GenBank accession numbers for Class II TPS9 proteins are as follows: XP_007133328.1 (*Pv*TPS1), XP_007149513.1 (*Pv*TPS2), XP_007159510.1 (*Pv*TPS3), XP_007142960.1 (*Pv*TPS4), XP_007153644.1 (*Pv*TPS5), XP_007157471.1 (*Pv*TPS6), XP_007154820.1 (*Pv*TPS7), XP_007155406.1 (*Pv*TPS8), XP_007153210.1 (*Pv*TPS9), XP_007138100.1 (*Pv*TPS10). **(B)** Relative expression levels of the *P. vulgaris* Class II TPS-encoding genes in 21 dpi (days post-inoculation) wild-type root nodules elicited by *R. etli* strain CFN42. The qPCR data came from six independent (*n* = 6) wild-type roots, tested in triplicate and normalized to the expression of the *Elongation factor 1-alpha* (*PvEF1a*) gene. Plotted data represent the expression ratio and are shown as means ± SD. **(C)** *PvTPS9* gene expression profile during nodule development. qPCR data came from six independent (*n* = 6) biological replicates, tested by technical triplicate and normalized to the expression of the *Elongation factor 1-alpha* (*PvEF1a*) reference gene. Plotted data represent the expression level of *PvTPS9* in nodules (closed squares) or in nodule-depleted roots (open squares) at different times (dpi), and are shown as means ± SD.

We identified 10 genes encoding trehalose-6-phosphate synthases (TPS) and 9 genes encoding trehalose 6-phosphate phosphatases (TPP) in the common bean genome (Phytozome v11, http://www.phytozome.net; Mazorka, http://mazorka.langebio.cinvestav.mx/blast/). Amino acid sequence comparisons allowed us to classify these proteins into the previously well-established Class I, Class II, and Class III gene families involved in the biosynthesis of trehalose (Leyman et al., 2001; Avonce et al., 2006). The common bean genome encoded three Class I genes (*PvTPS1-PvTPS3*), seven Class II genes (*PvTPS4-PvTPS10*) (Figure 1A and Table S2), and nine Class III *TPP* genes (*PvTPPA-PvTPPI*) (Table S2). Class I gene members were divided in two subgroups that diverged after the division between monocots and dicots. One subgroup contained only *PvTPS3*, its sequence being closer to *TPS* genes from other legume species such as *Lotus japonicus*; the other subgroup contained *PvTPS1* and *PvTPS2* (Figure 1A). As found in other plants, common bean Class II TPS proteins were clustered in subfamilies containing members from both monocots and dicots (Figure 1A), suggesting that the Class II TPS of common bean arose from ancient gene duplication events followed by specialization, as has been proposed for *Arabidopsis thaliana* Class II TPS (Ramon et al., 2009; Avonce et al., 2010). *In silico* analysis of the *P. vulgaris* transcriptome (Ramírez et al., 2005; Hernández et al., 2007; DFCI, http://compbio.dfci.harvard.edu/tgi/) revealed that all *TPS* and *TPP* genes in common bean plants were expressed, either during some part of plant development or in a tissue-specific manner.

We performed qRT-PCR assays using total RNA obtained from wild-type root nodules at 21 dpi to assess the accumulation of transcripts of common bean *TPS* genes. Results indicate that the *PvTPS9* transcript is the major transcript of the Class II *TPS* family members that accumulated in this organ (Figure 1B). Then, we specifically determined the expression profile of *PvTPS9* in roots and in root nodules of common bean plants at 14, 22, 24, and 28 dpi (Figure 1C). In nodule-depleted roots, the transcript level of *PvTPS9* remained practically constant from 14 to 22 dpi. However, in root nodules from 22 to 24 dpi, its accumulation decreased by 55 ± 2%, then returned to levels similar to those observed at 14 to 22 dpi. The transcript level of *PvTPS9* did not change in 14 to 24 dpi root nodules, although there was a significant increase from 24 to 28 dpi (Figure 1C), suggesting that *PvTPS9* plays an important role during the senescence of the root nodules.

### Silencing of *PvTPS9* diminishes trehalose levels and modifies the expression of other class II *TPS* family members in the symbiotic nodule

**Figure 2:**
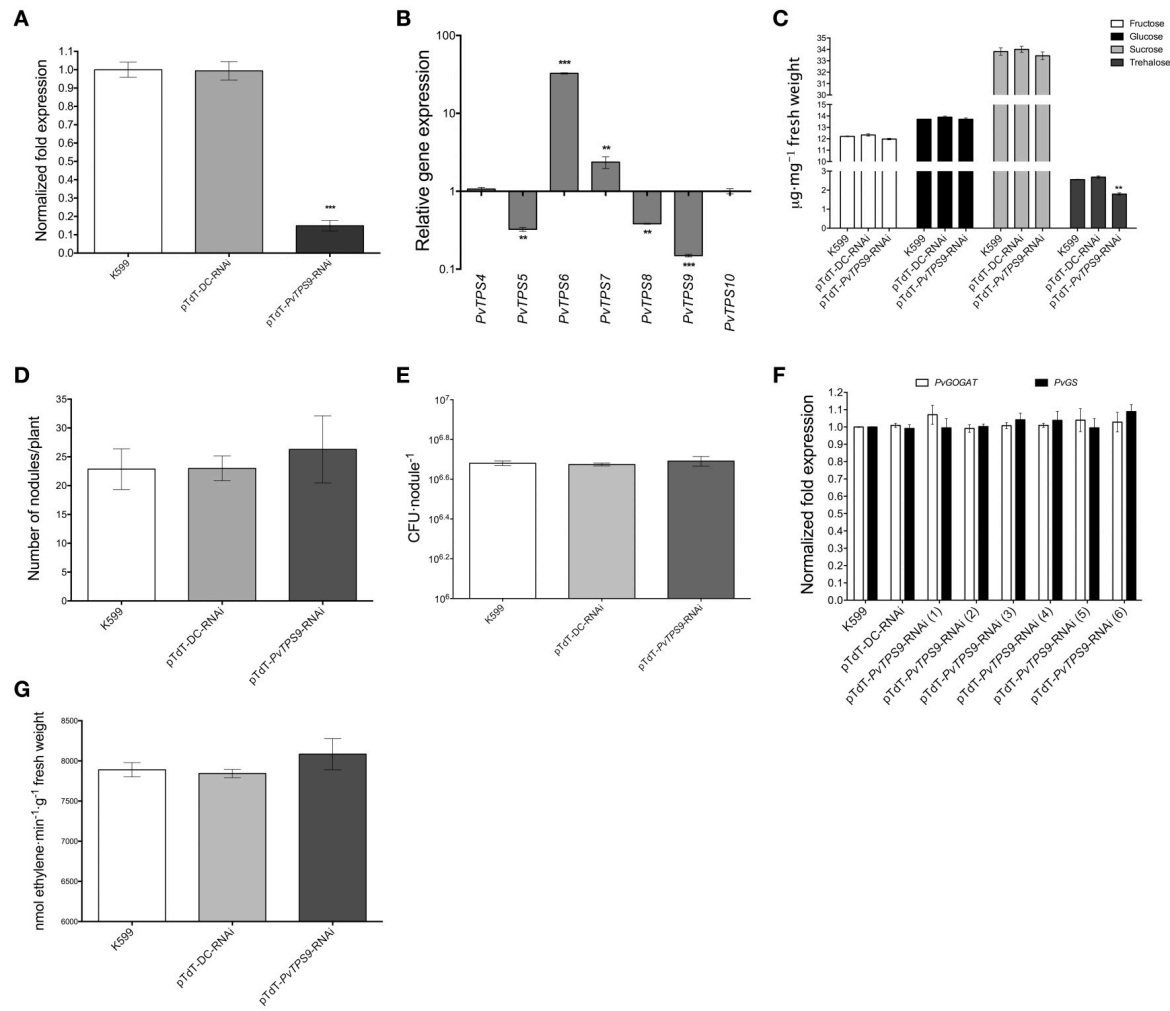
**Effects of *PvTPS9* down-regulation in 21 dpi transgenic root nodules from composite bean (*P. vulgaris*) plants. (A)** Down-regulation of *PvTPS9* in *PvTPS9*-RNAi nodules compared to control nodules (*A. rhizogenes* K599 or vector-transgenic roots) of the same age. qPCR data came from nodules of six independent (*n* = 6) biological replicates, tested by technical triplicates; the statistical significance was determined with an unpaired two-tailed Student's *t*-test (^*^^*^^*^P < 0.001), and shown as means ± SD. **(B)** Relative expression levels of the *P. vulgaris* Class II TPS-encoding genes in *PvTPS9*-RNAi nodules of composite bean plants. qPCR data came from nodules of six (*n* = 6) independent biological replicates, tested by technical triplicates and normalized to the expression of the *Elongation factor 1-alpha* (*PvEF1a*), as reference gene. Plotted data are the log_10_ of the expression of Class II *TPS* genes in control nodules and are shown as means ± SD. **(C)** Quantification of soluble carbohydrates (fructose, glucose, sucrose, and trehalose) in control nodules and in *PvTPS9*-RNAi nodules. The results presented for soluble carbohydrate quantification are means ± SD from three independent (*n* = 3) composite plants, and statistical significance was determined with one-way ANOVA followed by Dunnett's test (^*^^*^P < 0.01). Results are expressed as μg/mg of fresh weight. **(D)** The total number of nodules was scored in both, controls and in *PvTPS9*-RNAi transgenic roots of composite bean plants 21 dpi (*n* = 3), and statistical significance was determined with an unpaired two-tailed Student's *t*-test. No statistical significance was found. **(E)** *R. etli* strain CFN42 survival determined by colony-forming units (CFUs) re-isolated from control or *PvTPS9*-RNAi-nodules. The results presented for CFUs are means ± SD from nodules of three (*n* = 3) independent roots of composite plants, and statistical significance was determined with an unpaired two-tailed Student's *t*-test. No statistical significance was found. **(F)** Relative expression levels of *PvGOGAT* and *PvGS* in control and *PvTPS9*-RNAi nodules. The number in brackets indicates the independent transgenic roots from composite common bean plants analyzed. **(G)** Nitrogenase activity in *PvTPS9*-RNAi nodules. The results for nitrogenase activity are means ± SD from three independent (*n* = 3) composite roots. No statistical significance was found.

Previous work demonstrated the successful use of RNA interference (RNAi) to silence genes in composite bean plants, which have a wild-type shoot and transgenic roots (Valdés-López et al., 2008; Montiel et al., 2012; Barraza et al., 2013). Here, using the same approach mediated by *Agrobacterium rhizogenes* strain K599, we down-regulated the expression of *PvTPS9* by 85 ± 1% in 21 dpi transgenic nodules transformed with the pTdT-*PvTPS9-*RNAi construct (Figure 2A). In these experiments, nodules formed on hairy roots of common bean plants induced by untransformed *A. rhizogenes* K599 (used as a control) or by *A. rhizogenes* transformed with the pTdT-DC-RNAi (control nodules with empty vector) had unchanged levels of *PvTPS9* expression (Figure 2A).

Transcript levels of all Class II *TPS* family members of common bean were determined in pTdT-*PvTPS9-*RNAi 21 dpi transgenic root nodules by qPCR. Compared to control nodules [*A. rhizogenes* K599 or *A. rhizogenes* K599 transformed with the empty vector (pTd-DC-RNAi)], accumulation of *PvTPS9* transcript was reduced by 7-fold (Figure 2B). In these plants, the accumulation of *PvTPS6* and *PvTPS7* transcripts also decreased, by 33- and 2-fold, respectively, while *PvTPS4* and *PvTPS10* decreased by 3-fold (Figure 2B). *PvTPS1* and *PvTPS7* were the only members of this gene family for which expression was not altered (Figure 2B). However, in *PvTPS9* down-regulated nodules, the trehalose content declined up to 30 ± 3% (Figure 2C), which did not affect the final number of nodules (Figure 2D) or the number of bacteroids within these nodules (Figure 2E).

Interestingly, neither the silencing of *PvTPS9* or the changes in expression of other Class II *TPS* genes in root nodules modified their capacity to fix nitrogen, as deduced from the unaltered expression of the transcripts corresponding to two key enzymes involved in this process, NADH-Glutamate Synthase II (*PvGOGAT*) and Glutamine Synthetase (*PvGS*) (Figure 2F), as well as by the acetylene gas reduction rate (Figure 2G) observed in *PvTPS9* down-regulated nodules *versus* control nodules.

Together, these data suggest that directly or indirectly *Pv*TPS9 modulates trehalose levels in the nodule, but does not interfere with the nitrogen-fixation process.

### Silencing of *PvTPS9* in composite common bean roots dramatically affects nodule morphology and bacteroid size

**Figure 3:**
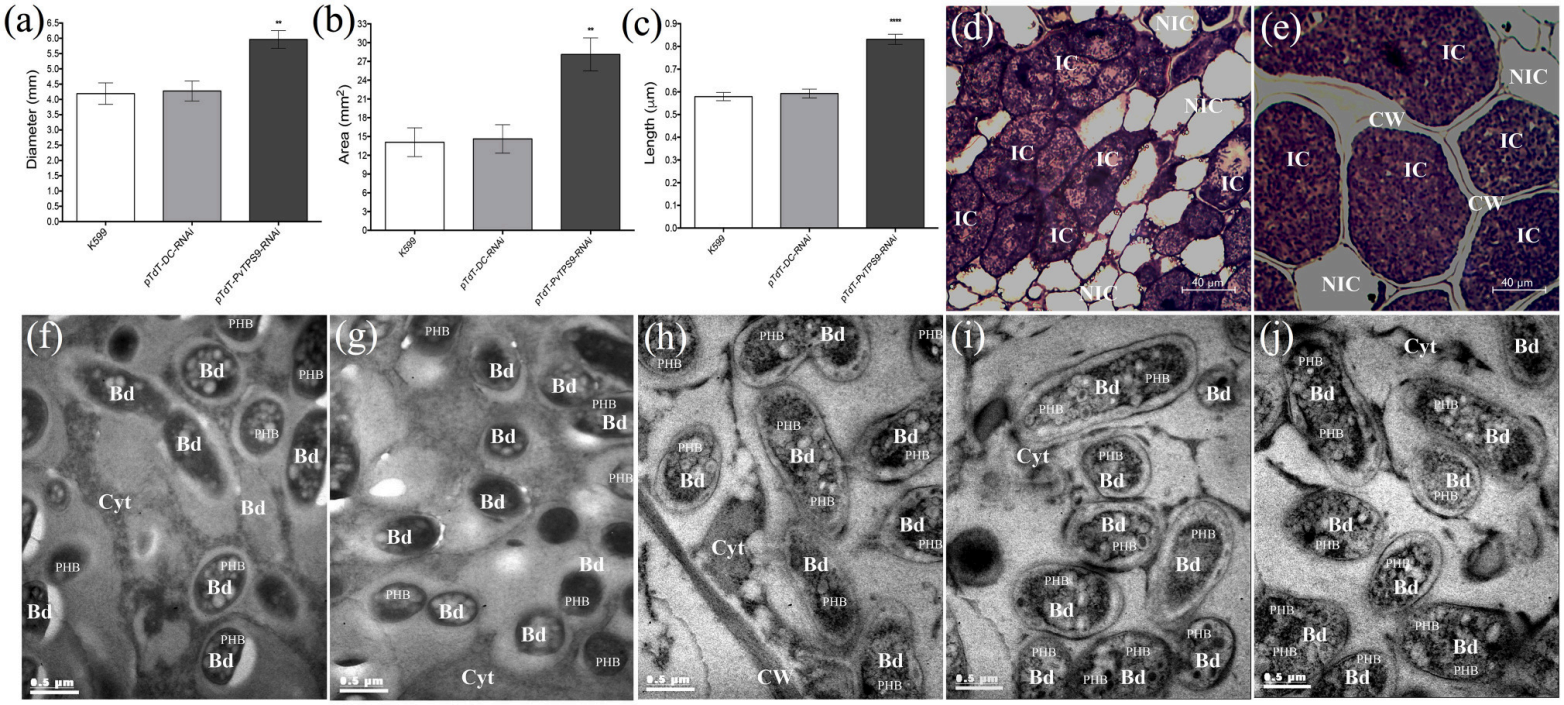
**Size and structure of *PvTPS9*-RNAi transgenic nodules in *Phaseolus vulgaris***. **(A)** Diameter and total area **(B)** measurements of control and *PvTPS9*-silenced nodules of composite bean plants. Data came from 27 nodules of each experimental condition. Results are means ± SD from three (*n* = 3) independent experiments, and statistical significance was determined with an unpaired two-tailed Student's *t*-test (^*^^*^P < 0.01). **(C)** Length of resident symbionts in control and *PvTPS9*-RNAi transgenic, infected cells. Data came from 9 (*n* = 9) independent transmission electron microscopy (TEM) samples and are direct measurements. Statistical significance was determined with an unpaired two-tailed Student's *t*-test (^*^^*^^*^^*^P < 0.001). **(D,E)** Optical microscopy of a pTdT-DC-RNAi **(D)** and a *PvTPS9*-RNAi (e) root nodule. **(F–J)** TEM of bacteroids in K599 **(F)**, empty vector **(G)**, and *PvTPS9*-RNAi nodules **(H–J)**. Representative results are shown. CW, cell wall; IC, infected cells; NIC, non-infected cells; Bd, bacteroids; Cyt, cytoplasm; PHB, poly-β–hydroxybutyrates.

Determinate common bean root nodules consist of three major tissues: a central infection zone (mainly composed by infected and uninfected cells), an inner cortex that includes vascular bundles, and an outer cortex. All of our silenced and control plants produced similar final numbers of viable bacteroids within the nodules (Figure 2E), and similar numbers of nodules per plant (Figure 2D). By contrast, the nodule size in pTdT-*PvTPS9-*RNAi nodules dramatically increased (44 ± 4%, Figures 3A,B). Detailed observations of nodule sections stained with toluidine blue (Figures 3D,E) indicated a notable increase in the thickness of the cell wall in both infected and uninfected cells (Figure 3E) compared to controls (Figure 3D). Moreover, both infected and uninfected cells of pTdT-*PvTPS9-*RNAi nodules (Figure 3E) were larger than the cells in control nodules (Figure 3D).

Ultra-thin sections of 21 dpi pTdT-*PvTPS9-*RNAi transgenic nodules as well as nodules from control roots (*A. rhizogenes* strain K599 and empty vector) were analyzed by transmission electron microscopy (TEM, Figures 3F,J). Compared to controls (Figures 3F,G,**C**), bacteroids contained in pTdT-*PvTPS9-*RNAi nodules were considerably larger (Figures 3H,J,**C**). These bacteroids accumulated higher amounts of poly-β-hydroxybutyrate (PHB) in the cytoplasm (Figures 3F,G compared to Figures 3H–J), a carbon polymer product of glucose or starch assimilation employed by rhizobia as an energy storage molecule that can be metabolized when other common energy sources are not available.

As the plant carbohydrate supply has been proposed to regulate nodule development, we explored whether differences in the soluble carbohydrate content of *PvTPS9-*RNAi and control nodules could explain the morphological variations observed in pTdT-*PvTPS9-*RNAi nodules. Remarkably, only trehalose showed a significant reduction in *PvTPS9*-RNAi nodules compared to controls.

### Silencing *PvTPS9* in root nodules produces a deleterious, systemic effect in composite bean plants

**Figure 4:**
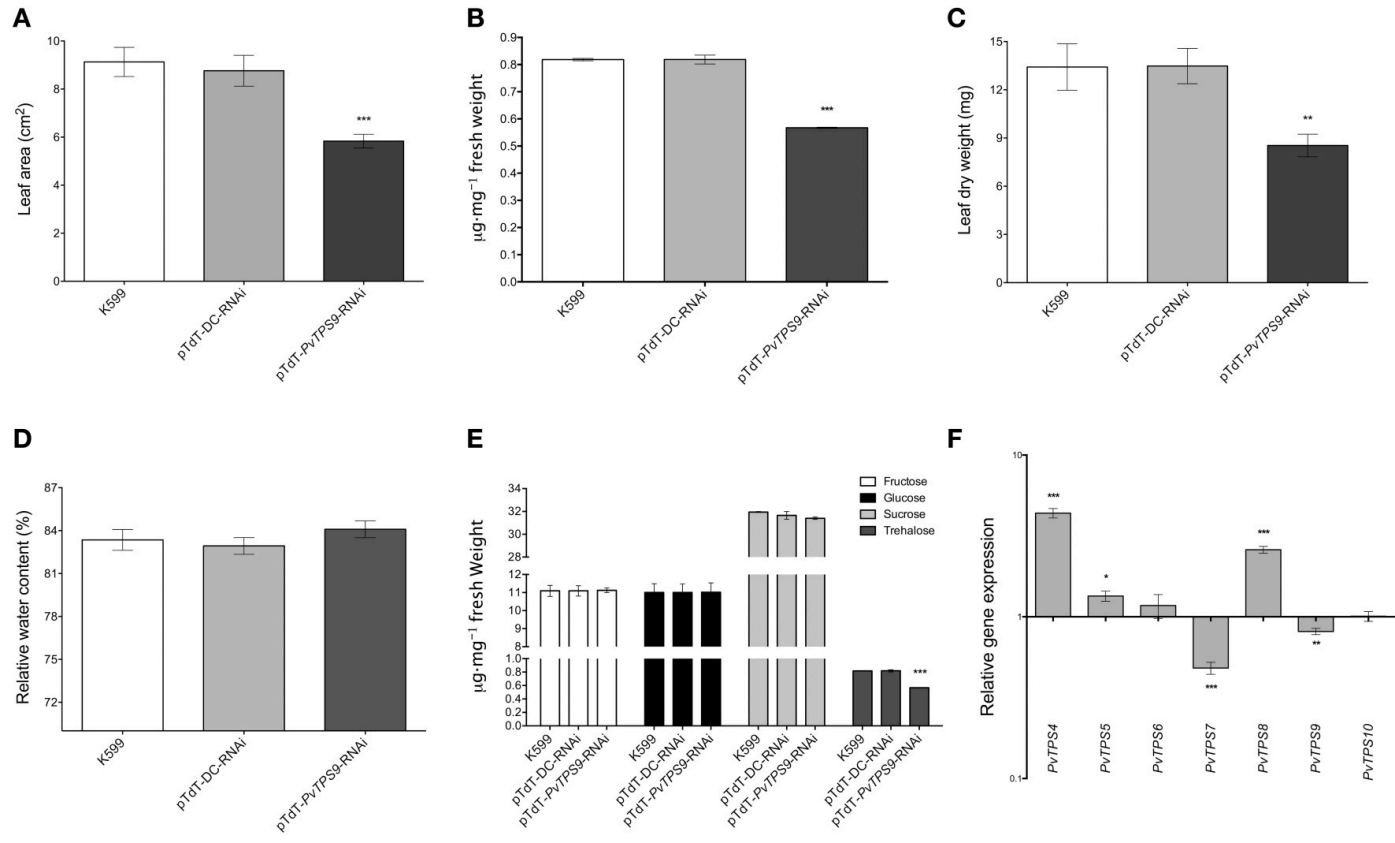
**Effect on leaves of *P. vulgaris PvTPS9*-RNAi transgenic roots. (A)** Leaf area of composite plants. The results for leaf area are means ± SD from three independent (*n* = 3) composite plants and statistical significance was determined with an unpaired two-tailed Student's *t*-test (^*^^*^^*^P < 0.001). **(B)** Trehalose content determination in leaves of *PvTPS9*-RNAi composite common bean plants. Results are means ± SD from three independent (*n* = 3) composite plants and statistical significance was determined with an unpaired two-tailed Student's *t*-test (^*^^*^^*^P < 0.001). **(C)** Leaf biomass determination. Dry leaf weights were obtained from three independent (*n* = 3) composite common bean plants. The results are means ± SD and statistical analysis was determined with an unpaired two-tailed Student's *t*-test (^*^^*^P < 0.01). **(D)** Relative water content (RWC) of leaves of composite plants. The results for RWC are means ± SD from three independent (*n* = 3) composite plants. No statistical significance was found with an unpaired two-tailed Student's *t*-test. **(E)** Soluble carbohydrate (fructose, glucose, sucrose, and trehalose) quantification in leaves of composite plants. The results presented for soluble carbohydrate quantification in leaves of composite plants are means ± SD from three independent (*n* = 3) composite plants, and statistical significance was determined with one-way ANOVA followed by Dunnett's test (^*^^*^^*^P < 0.001). **(F)** Relative gene expression levels of the *P. vulgaris* Class II TPS-encoding genes in 21 dpi (days post-inoculation) composite common bean plants. qPCR data came from the foliage of three independent composite plants (*n* = 3) tested by technical triplicate and normalized to the expression of the *Elongation factor 1–alpha* (*PvEF1a*) gene, and statistical significance was determined with an unpaired two-tailed Student's *t*-test (^*^P < 0.05; ^*^^*^P < 0.01; ^*^^*^^*^P < 0.001). Plotted data are expressed as log_10_ of the relative gene expression of control Class II *TPS* genes, and are shown as means ± SD.

In pTdT-*PvTPS9-*RNAi composite common bean plants inoculated with *R. etli* strain CFN42, we observed a clear reduction in leaf size compared to inoculated controls (Figure S2). We quantified the leaf area (Figure 4A) and the leaf biomass (Figures 4B,C) in these plants, and found reductions of 36 ± 3% and 36 ± 5%, respectively. However, the foliage of pTdT-*PvTPS9-*RNAi composite bean plants did not show chlorosis (Figure S2), a common symptom in plants subjected to environmental stresses. The reduction in leaf size of pTdT-*PvTPS9-*RNAi composite common bean plants was not caused by water deficit, based on comparisons of the relative water content of pTdT-*PvTPS9-*RNAi- and control plants (Figure 4D).

Remarkably, analysis of the trehalose content in leaves of pTdT-*PvTPS9-*RNAi composite plants indicated a 30% reduction of this carbohydrate compared to control plants (Figure 4E), similar to that observed in root nodules (Figure 2C). Unexpectedly, other soluble sugars such as sucrose, glucose and fructose, did not show any change (Figure 4E). It has been reported that Class II TPS gene expression responds to carbon availability in *A. thaliana* (Ramon et al., 2009). Accordingly, we determined the transcript levels of the common bean Class II *TPS* gene family members, and found that *PvTPS4, PvTPS5*, and *PvTPS8* were up-regulated by 4-fold, 1-fold, and 3-fold, respectively (Figure 4F), whereas *PvTPS7* and *PvTPS9* were down-regulated by 2.1-fold and 1.2-fold (Figure 4F). The remaining Class II *TPS* gene family members, *PvTPS6* and *PvTPS10*, did not significantly change in expression (Figure 4F).

## Discussion

In this work we used an *in silico* approach to identify trehalose biosynthesis-related genes in the genome of common bean (*P. vulgaris* L.) and found 10 TPS- and 9 TPP-encoding genes (Table S2). A similar number is found in other plant species (Avonce et al., 2010; Vandesteene et al., 2012). In *P. vulgaris*, three of the *TPS* genes belong to Class I (*PvTPS1-3*), whereas the other seven belong to Class II (*PvTPS4-10*) (Table S2). As in *A. thaliana*, phylogenetic analysis of the *TPS* genes of common bean revealed pairs of paralogous loci, such as *PvTPS1* and *PvTPS2, PvTPS5* and *PvTPS6*, or *PvTPS8*, and *PvTPS10*. These paralogs have been proposed to be the result of a recent whole-genome duplication event (Figure 1A). By contrast, other genes, like *PvTPS9* or *PvTPS7*, are grouped in clades formed by monocot or dicot species (Figure 1A), suggesting their presence in a common ancestor (Avonce et al., 2010; Vandesteene et al., 2012; Schmutz et al., 2014; Vlasova et al., 2016).

In common bean plants, all the *TPS* genes were expressed either in different tissues or in different developmental stages (Ramírez et al., 2005; Hernández et al., 2007, DFCI, http://compbio.dfci.harvard.edu/tgi/). Nevertheless, *PvTPS9* was the most abundantly expressed *TPS* gene in functional nitrogen-fixing root nodules (Figure 1B), a result also seen in other studies (Ramírez et al., 2005; Hernández et al., 2007). Moreover, the *PvTPS9* pattern of transcript accumulation during nodule development, compared to its expression in roots (Figure 1C) strongly suggests that *PvTPS9* plays an important role in nodule function. Interestingly, several reports have shown that, although Class II TPS proteins contain TPS and TPP domains, they do not possess the corresponding enzymatic activities (Vogel et al., 2001; Ramon et al., 2009; Avonce et al., 2010; Vandesteene et al., 2012; Lunn et al., 2014).

To evaluate the possible role of this protein during root nodule symbiosis, we silenced the expression of *PvTPS9* in roots of composite common bean plants infected with *R. etli* strain CFN42. Compared to controls, *PvTPS9*-RNAi nodules showed a reduction in *PvTPS9* transcript levels below 80% (Figure 2A) and a change in expression of most Class II *TPS* gene family members (Figure 2B). This variation in gene expression coincided with a significant decrease in the nodule trehalose content (Figure 2C). Class II *TPS* gene expression responds to carbon availability in *A. thaliana* (Ramon et al., 2009). Our data from *PvTPS9-*RNAi composite common bean plants, in which the expression of most of the Class II *TPS* family members has been modified, further indicate that Class II *TPS* genes could have important roles in carbon allocation.

Remarkably, a similar effect was observed in the foliage of pTdT-*PvTPS9*-RNAi composite common bean plants inoculated with *R. etli* strain CFN42 (Figures 4E,F), which displayed nearly a 40% reduction in leaf size and leaf biomass compared to controls (Figures 4A–D and Figure S2). This is not the first time that organ-specific manipulation of trehalose metabolism has affected the whole plant. We previously demonstrated that silencing the expression of trehalase (*PvTRE1*) in root nodules of composite common bean plants positively influences the rate of nitrogen fixation by increasing the number of active bacteroids in transformed nodules by approx. 10-fold, which induces a significant increase in the trehalose content in nodules and in the foliage (Barraza et al., 2013). Trehalose and associated metabolites are important systemic modulators of plant growth and sugar signaling (Gómez et al., 2006, 2010; Schluepmann and Paul, 2009; Wingler et al., 2012; Lunn et al., 2014). Therefore, although we did not observe any change in metabolizable sugars in *PvTPS9*-RNAi composite common plants (Figures 2C, 4E), the reduction of trehalose may be responsible for altering the metabolism of carbon in the whole plant, thus reducing plant growth.

In this work we showed that *PvTPS9*-RNAi nodules, which on average contained 30% less trehalose (Figure 2C), were 33% larger (Figures 3A,B) and displayed important structural modifications, such as the enlargement of both infected and uninfected cells, and a remarkable thickening of the cell wall (Figures 3D,E). Furthermore, resident bacteroids of these organs were considerably larger than their equivalents in control nodules and contained a higher number of poly-β-hydroxybutyrate (PHB) granules in their cytoplasm (Figures 3H–J compared to Figures 3C, F, G). Although, the precise roles of PHB metabolism in the legume-rhizobium symbiosis are not completely understood, experimental evidence indicates that PHB is biosynthesized and turned over rapidly during active nitrogen fixation, and is extremely important for rhizobia to regulate plant carbon flux (Lodwig and Poole, 2003; Trainer et al., 2010). Even though the nitrogen fixation rate in *PvTPS9*-RNAi nodules was not changed (Figure 2G), their altered morphology suggests that the metabolism of trehalose plays a pivotal role in nodule formation and development, and affects the carbon metabolism in rhizobia.

Different lines of evidence suggest that most of the trehalose accumulated in the nodule originates from the bacteria, rather than from the plant (Müller et al., 2001; Suárez et al., 2008; López et al., 2009; Vauclare et al., 2010). Thus, the changes observed in the concentration of trehalose in composite *PvTPS9*-RNAi and control plants could be explained by an adjustment in the biosynthesis of trehalose by rhizobia in response to changes in the carbon metabolism of the plant caused by variations in the expression of the plant Class II *TPS* genes. Although, *PvTPS9* encodes a Class II TPS, a well-known non-catalytically active TPS or TPP protein (Ramon et al., 2009; Vandesteene et al., 2012; Lunn et al., 2014), our data indicate that *Pv*TPS9 plays a role in the regulation of trehalose metabolism in the symbiotic nodule and in consequence, of the whole plant.

*VuTPS6*, a Class II TPS gene from *Vigna unguiculata*, has been reported to be regulated by the miRNA *Vu*miR172b (Lu and Yang, 2010). Interestingly, *Pv*miR172, a homolog of *Vu*miR172, is abundant during common bean nodulation (Peláez et al., 2012 and Figure S4B of this work). Excitingly, a sequence alignment of *Pv*miR172 and the transcript of *PvTPS9*, revealed a possible recognition site in the coding region (Figure S4A). The transcript abundance of the canonical target of miR172, *APETALA2* (*PvAP2*), shows an inverse correlation to that of *Pv*miR172 during nodulation, an indication that *Pv*miR172 modulates the expression of *PvAP2* in *P. vulgaris* (Mlotshwa et al., 2006; Glazińska et al., 2009; Wollmann et al., 2010; Grigorova et al., 2011; Zhu and Helliwell, 2011; Varkonyi-Gasic et al., 2012). Here, we found that the *PvTPS9* transcript was associated with *Pv*AGO1-complexes from 14 and 22 dpi nodules along with *PvAP2* and *Pv*miR172, despite the fact that 5′-RACE analysis revealed no evidence of processing for the *PvTPS9* transcript (Figure S4C). In contrast to *PvAP2*, however, *PvTPS9* levels showed a direct (rather than inverse) correlation with those of *Pv*miR172 (Figure S4B), suggesting there is no direct regulation of *PvTPS9* by *Pv*miR172 via mRNA degradation. This miRNA may affect *PvTPS9* by another type of regulation, such as inhibition of translation (Voinnet, 2009; Rüegger and Grosshans, 2012). Still, in p35S::*ReTPS* and *PvTRE1*-RNAi transgenic nodules, where the trehalose content increased, we also observed a significant reduction in the *Pv*miR172 abundance (Figure S4D). This behavior is opposite to that in *PvTPS9*-RNAi nodules (Figure S5), suggesting that the trehalose content has a direct effect on the abundance of *Pv*miR172. Interestingly, miR172 modulates nodule organogenesis in leguminous plants (Wang et al., 2014; Holt et al., 2015; Nova-Franco et al., 2015).

We also monitored *Pv*miR172 and *PvAP2* transcript abundance in the foliage of *PvTPS9*-RNAi composite common bean plants (Figures S3, S5). Here also, the level of *Pv*miR172 increased whereas the level of *PvAP2* transcript decreased (Figures S3, S5), supporting the idea that the trehalose content modulates the expression of *Pv*miR172 *in planta*.

In conclusion, we propose that *Pv*TPS9 plays a key role in modulating trehalose metabolism in the symbiotic nodule and, therefore, in the whole plant. Through such control, *Pv*TPS9 influences nodule and plant development. Although, we found no evidence for direct regulation of *PvTPS9* by *Pv*miR172, the fact that the trehalose content in symbiotic nodules alters *Pv*miR172 abundance strongly suggests that *Pv*TPS9 is indirectly involved in regulating *Pv*miR172 expression. It is tempting to speculate that *Pv*miR172 regulates *Pv*TPS9 through translation inhibition, forming a feedback regulatory loop.

## Author contributions

AB was responsible for the experimental work and made substantial contributions to conceptualization and design of the study. Furthermore, he was involved in drafting the manuscript. CC, GE, MV, and JR made substantial contributions to the experimental work. JR, NA, CQ, FS, and CD made substantial contributions to conceptualization and design of the study and were involved in drafting the manuscript and revising it critically for important intellectual content. FS and CD contributed reagents/materials/analysis and CQ and CD gave final approval for the version to be published.

## Funding

AB was supported by a PhD scholarship (169219) from Consejo Nacional de Ciencia y Tecnología (CONACYT)-México. This research was partially supported by grants from CONACYT-México (No. 177207) and from the Universidad Nacional Autónoma de México (Dirección General Asuntos del Personal Académico PAPIIT No. IN206815) to CD.

### Conflict of interest statement

The authors declare that the research was conducted in the absence of any commercial or financial relationships that could be construed as a potential conflict of interest.

## Supplementary material

Figure S1 **Schematic representation of the *PvTPS9* cDNA sequence**. The coding sequence (CDS) and the 3′untranslated region is shown in white boxes, and the DNA fragment amplified by qPCR and the target region to be silenced by RNAi are shown in green and red boxes, respectively. We also indicate the primer sequence and position to measure *PvTPS9* transcript abundance by qPCR and to amplify the 300 bp DNA sequence used in our RNAi design.

Figure S2 **Control (*A. rhizogenes* K599) or *PvTPS9-*RNAi composite bean plants**. Photograph was taken at 21 dpi.

Figure S3 ***Pv*miR172, *PvAP2*, and *PvTPS9* transcript abundance determined in the different tissues of common bean plants by qPCR assays**. Data came from plant tissues (roots, nodules, leaves, seeds and pods) of six independent (*n* = 6) biological replicates and shown as means ± SD.

Figure S4 ***Pv*miR172 expression could be regulated systemically by the trehalose content in *P. vulgaris* plants. (A)** Schematic representation of the transcript sequence of *PvTPS9* and the putative interaction with *Pv*miR172, showing three mismatches near the central zone of *PvTPS9*. **(B)** *Pv*miR172 (circles), *PvAP2* (triangles), *PvTPS9* (squares) gene expression profile during nodule development. qPCR data came from six independent (*n* = 6) wild-type nodules, tested by triplicate and normalized to the expression level of the *Elongation factor 1-alpha* (*PvEF1a*) gene. Plotted data are shown as mean ± SD. **(C)** Anti-*Pv*AGO1 RNA co-immunoprecipitation (IP). Total extracts of 14 or 21 dpi wild-type nodules were divided in four parts and used for anti-*Pv*AGO1 immunoprecipitation followed by RNA extraction and cDNA synthesis. qPCR data came from three (*n* = 3) independent experimental replicas tested by triplicate and normalized to the *Pv*miR2118 reference gene (Livak and Schmittgen, 2001). **(D)** *Pv*miR172 abundance and trehalose content in control (*A. rhizogenes* K599) or in p35S::*ReTPS, PvTPS9*-RNAi and *PvTRE1*-RNAi transgenic nodules of composite bean plants. qPCR data came from nodules of six independent (*n* = 6) transgenic roots. The statistical signficance was determined with an unpaired two-tailed Student's *t*-test (^*^*P* < 0.05, ^**^*P* < 0.01, ^***^*P* < 0.001), and shown as means ± SD.

Figure S5 ***Pv*miR172 and *PvAP2* transcript abundance in leaves of *PvTPS9*-RNAi composite common bean plants by qPCR assays. (A,B)** Statistical significance was determined with an unpaired two-tailed Student's *t*-test (^*^*P* < 0.05; ^***^*P* < 0.001). Plotted data are expressed as log_10_ of relative gene expression of Class II *TPS* genes in the control composite common bean plants and shown as means ± SD.

Table S1 **List of primers used in this work**.

Table S2 **Class I, Class II, and Class III gene families involved in the biosynthesis of trehalose in common bean (Phytozome v11, http://www.phytozome.net; Mazorka, http://mazorka.langebio.cinvestav.mx/blast/)**.

## References

[B1] Arenas-Huertero C. Pérez B. Rabanal F. Blanco-Melo D. De la Rosa C. Estrada-Navarrete G.. (2009). Conserved and novel miRNAs in the legume *Phaseolus vulgaris* in response to stress. Plant Mol. Biol. 70, 385–401. 10.1007/s11103-009-9480-3 19353277

[B2] Avonce N. Mendoza-Vargas A. Morett E. Iturriaga G. (2006). Insights on the evolution of trehalose biosynthesis. BMC Evol. Biol. 6:109. 10.1186/1471-2148-6-109 17178000 PMC1769515

[B3] Avonce N. Wuyts J. Verschooten K. Vandesteene L. Van Dijck P. (2010). The Cytophaga hutchinsonii ChTPSP: first characterized bifunctional TPS-TPP protein as putative ancestor of all eukaryotic trehalose biosynthesis proteins. Mol. Biol. Evol. 27, 359–369. 10.1093/molbev/msp241 19812028

[B4] Bacanamwo M. Harper J. E. (1996). Regulation of nitrogenase activity in *Bradyrhizobium japonicum*/soybean symbiosis by plant N status as determined by shoot C:N ratio. Physiol. Plant. 98, 529–538. 10.1111/j.1399-3054.1996.tb05708.x

[B5] Bacanamwo M. Harper J. E. (1997). The feedback mechanism of nitrate inhibition of nitrogenase activity in soybean may involve asparagine and/or products of its metabolism. Physiol. Plant. 100, 371–377. 10.1007/s12020-016-0874-0 27351066

[B6] Barraza A. Estrada-Navarrete G. Rodriguez-Alegria M.-E. Lopez-Munguia A. Merino E. Quinto C.. (2013). Down-regulation of PvTRE1 enhances nodule biomass and bacteroid number in the common bean. New Phytol. 197, 194–206. 10.1111/nph.12002 23121215

[B7] Brodmann D. Schuller A. Ludwig-Müller J. Aeschbacher R. A. Wiemken A. Boller T.. (2002). Induction of trehalase in Arabidopsis plants infected with the trehalose-producing pathogen Plasmodiophora brassicae. Mol. Plant-Microbe Interact. 15, 693–700. 10.1094/MPMI.2002.15.7.693 12118885

[B8] Broughton W. J. Dilworth M. J. (1971). Control of leghaemoglobin synthesis in snake beans. Biochem. J. 125, 1075–1080. 10.1042/bj1251075 5144223 PMC1178271

[B9] Contreras-Cubas C. Rabanal F. A. Arenas-Huertero C. Ortiz M. A. Covarrubias A. A. Reyes J. L. (2012). The *Phaseolus vulgaris* miR159a precursor encodes a second differentially expressed microRNA. Plant Mol. Biol. 80, 103–115. 10.1007/s11103-011-9847-0 22083131

[B10] Estrada-Navarrete G. Alvarado-Affantranger X. Olivares J.-E. Guillén G. Díaz-Camino C. Campos F.. (2007). Fast, efficient and reproducible genetic transformation of Phaseolus spp. By *Agrobacterium rhizogenes*. Nat. Protoc. 2, 1819–1824. 10.1038/nprot.2007.259 17641650

[B11] Ferguson B. J. Indrasumunar A. Hayashi S. Lin M.-H. Lin Y.-H. Reid D. E.. (2010). Molecular analysis of legume nodule development and autoregulation. J. Integr. Plant Biol. 52, 61–76. 10.1111/j.1744-7909.2010.00899.x 20074141

[B12] Figueroa C. M. Feil R. Ishihara H. Watanabe M. Kölling K. Krause U.. (2016). Trehalose 6-phosphate coordinates organic and amino acid metabolism with carbon availability. Plant J. 85, 410–423. 10.1111/tpj.13114 26714615

[B13] Figueroa C. M. Lunn J. E. (2016). A tale of two sugars: trehalose 6-phosphate and sucrose. Plant Physiol. 172, 7–27. 10.1104/pp.16.00417 27482078 PMC5074632

[B14] Foster A. J. Jenkinson J. M. Talbot N. J. (2003). Trehalose synthesis and metabolism are required at different stages of plant infection by *Magnaporthe grisea*. EMBO J. 22, 225–235. 10.1093/emboj/cdg018 12514128 PMC140093

[B15] Gage D. J. (2004). Invasion and infection of roots by symbiotic, nitrogen-fixing rhizobia during nodulation of temperate legumes. Microbiol. Mol. Biol. Rev. 68, 280–300. 10.1128/MMBR.68.2.280-300.2004 15187185 PMC419923

[B16] Galtier N. Gouy M. Gautier C. (1996). SEAVIEW and PHYLO_WIN: two graphic tools for sequence alignment and molecular phylogeny. Comput. Appl. Biosci. 12, 543–548. 10.1093/bioinformatics/12.6.543 9021275

[B17] Garg N. Singla P. (2016). Stimulation of nitrogen fixation and trehalose biosynthesis by naringenin (Nar) and arbuscular mycorrhiza (AM) in chickpea under salinity stress. Plant Growth Regul. 80, 5–22. 10.1007/s10725-016-0146-2

[B18] Gibson A. H. Harper J. E. (1985). Nitrate effect on nodulation of soybean by *Bradyrhizobium japonicum*. Crop Sci. 25, 497–501. 10.2135/cropsci1985.0011183X002500030015x

[B19] Glazińska P. Zienkiewicz A. Wocjciechowski W. Kopcewicz J. (2009). The putative miR172 target gene InAPETALA2-like is involved in the photoperiodic flower induction of *Ipomea nil*. J. Plant Physiol. 166, 1801–1813. 10.1016/j.jplph.2009.05.011 19560230

[B20] Gómez L. D. Baud S. Gilday A. Li Y. Graham I. A. (2006). Delayed embryo development in the ARABIDOPSIS TREHALOSE-6-PHOSPHATE SYNTHASE 1 mutant is associated with altered cell wall structure, decreased cell division and starch accumulation. Plant J. 46, 69–84. 10.1111/j.1365-313X.2006.02662.x 16553896

[B21] Gómez L. D. Gilday A. Feil R. Lunn J. E. Graham I. A. (2010). AtTPS1-mediated trehalose 6-phosphate synthesis is essential for embryogenic and vegetative growth and responsiveness to ABA in germinating seeds and stomatal guard cells. Plant J. 64, 1–13. 10.1111/j.1365-313X.2010.04312.x 20659274

[B22] Grigorova B. Mara C. Hollender C. Sijacic P. Chen X. Liu Z. (2011). LEUNIG and SEUSS co-repressors regulate miR172 expression in Arabidopsis flowers. Development 138, 2451–2456. 10.1242/dev.058362 21610026 PMC4074289

[B23] Guindon S. Dufayard J. F. Lefort V. Anisimova M. Hordijk W. Gascuel O. (2010). New algorithms and methods to estimate maximum-likelihood phylogenies: assessing the performance of PhyML 3.0. Syst. Biol. 59, 307–321. 10.1093/sysbio/syq010 20525638

[B24] Hernández G. Ramírez M. Váldes-López O. Tesfaye M. Graham M. A. Czechowski T.. (2007). Phosphorus stress in common bean: root transcript and metabolic responses. Plant Physiol. 144, 752–767. 10.1104/pp.107.096958 17449651 PMC1914166

[B25] Holt D. B. Gupta V. Meyer D. Abel N. B. Andersen S. U. Stougaard J.. (2015). micro RNA 172 (miR172) signals epidermal infection and is expressed in cells primed for bacterial invasion in *Lotus japonicus* roots and nodules. New Phytol. 208, 241–256. 10.1111/nph.13445 25967282

[B26] Imsande J. (1986). Inhibition of nodule development in soybean by nitrate or reduced nitrogen. J. Exp. Bot. 37, 348–355. 10.1073/pnas.1607507113 27690404

[B27] Kouchi H. Imaizumi-Anraku H. Hayashi M. Hakoyama T. Nakagawa T. Umehara Y.. (2010). How many peas in a pod? Legume genes responsible for mutualistic symbiosis underground. Plant Cell Physiol. 51, 1381–1397. 10.1093/pcp/pcq107 20660226 PMC2938637

[B28] Leyman B. Van Dijck P. Thevelein J. M. (2001). An unexpected plethora of trehalose biosynthesis genes in *Arabidopsis thaliana*. Trends Plant Sci. 6, 510–513. 10.1016/S1360-1385(01)02125-2 11701378

[B29] Limpens E. Bisseling T. (2003). Signaling in symbiosis. Curr. Opin. Plant Biol. 6, 343–350. 10.1016/S1369-5266(03)00068-2 12873529

[B30] Livak K. J. Schmittgen T. D. (2001). Analysis of relative gene expression data using real-time quantitative PCR and the 2^−ΔΔCT^ method. Methods 25, 402–408. 10.1006/meth.2001.1262 11846609

[B31] Lodwig E. Poole P. (2003). Metabolism of Rhizobium bacteroids. CRC. Crit. Rev. Plant Sci. 22, 37–78. 10.1080/713610850

[B32] López M. Tejera N. A. Lluch C. (2009). Validamycin A improves the response of *Medicago truncatula* plants to salt stress by inducing trehalose accumulation in the root nodules. J. Plant Physiol. 166, 1218–1222. 10.1016/j.jplph.2008.12.011 19232773

[B33] Lu Y. Yang X. (2010). Computational identification of novel microRNAs and their targets in *Vigna unguiculata*. Comp. Funct. Genomics 2010:128297 10.1155/2010/128297 PMC2929582 20811611

[B34] Lunn J. E. Delorge I. Figueroa C. M. Van Dijck P. Stitt M. (2014). Trehalose metabolism in plants. Plant J. 79, 544–567. 10.1111/tpj.12509 24645920

[B35] Mlotshwa S. Yang Z. Kim Y. Chen X. (2006). Floral patterning defects induced by Arabidopsis APETALA2 and microRNA172 expression in *Nicotiana benthamiana*. Plant Mol. Biol. 61, 781–793. 10.1007/s11103-006-0049-0 16897492 PMC3574581

[B36] Montiel J. Nava N. Cárdenas L. Sánchez-López R. Arthikala M. K. Santana O.. (2012). A *Phaseolus vulgaris* NADPH oxidase gene is required for root infection by Rhizobia. Plant Cell Physiol. 53, 1751–1767. 10.1093/pcp/pcs120 22942250

[B37] Müller J. Boller T. Wiemken A. (2001). Trehalose becomes the most abundant non-structural carbohydrate during senescence of soybean nodules. J. Exp. Bot. 52, 943–947. 10.1093/jexbot/52.358.943 11432911

[B38] Nehls U. (2008). Mastering ectomycorrhizal symbiosis: the impact of carbohydrates. J. Exp. Bot. 59, 1097–1108. 10.1093/jxb/erm334 18272925

[B39] Nova-Franco B. Íñiguez L. P. Valdés-López O. Alvarado-Affantranger X. Leija A. Fuentes S. I.. (2015). The microRNA172c-APETALA2-1 node as a key regulator of the common bean-Rhizobium etli nitrogen fixation symbiosis. Plant Physiol. 168, 273–291. 10.1104/pp.114.255547 25739700 PMC4424015

[B40] Ocón A. Hampp R. Requena N. (2007). Trehalose turnover during abiotic stress in arbuscular mycorrhizal fungi. New Phytol. 174, 879–891. 10.1111/j.1469-8137.2007.02048.x 17504469

[B41] O'Rourke J. A. Iniguez L. P. Fu F. Bucciarelli B. Miller S. S. Jackson S. A.. (2014). An RNA-Seq based gene expression atlas of the common bean. BMC Genomics 15:866. 10.1186/1471-2164-15-866 25283805 PMC4195886

[B42] Peláez P. Trejo M. S. Iñiguez L. P. Estrada-Navarrete G. Covarrubias A. A. Reyes J. L.. (2012). Identification and characterization of microRNAs in *Phaseolus vulgaris* by high-throughput sequencing. BMC Genomics 13:83. 10.1186/1471-2164-13-83 22394504 PMC3359237

[B43] Pontius J. U. Wagner L. Schuler G. D. (2003). UniGene: an unified view of the transcriptome, in The NCBI Handbook, eds McEntyre J. Ostell J. (Bethesda, MD: National Center for Biotechnology Information), 1–11.

[B44] Qi Y. Mi S. (2010). Purification of Arabidopsis argonaute complexes and associated small RNAs. Methods Mol. Biol. 592, 243–254. 10.1007/978-1-60327-005-2_16 19802600

[B45] Ramírez M. Graham M. A. Blanco-López L. Silvente S. Medrano-Soto A. Blair M. W.. (2005). Sequencing and analysis of common bean ESTs. Building a foundation for functional genomics. Plant Physiol. 137, 1211–1227. 10.1104/pp.104.054999 15824284 PMC1088315

[B46] Ramon M. De Smet I. Vandesteene L. Naudts M. Leyman B. Van Dijck P.. (2009). Extensive expression regulation and lack of heterologous enzymatic activity of the Class II trehalose metabolism proteins from *Arabidopsis thaliana*. Plant Cell Environ. 32, 1015–1032. 10.1111/j.1365-3040.2009.01985.x 19344332

[B47] Ronquist F. Huelsenbeck J. P. (2003). MrBayes 3: Bayesian phylogenetic inference under mixed models. Bioinformatics 19, 1572–1574. 10.1093/bioinformatics/btg180 12912839

[B48] Rüegger S. Grosshans H. (2012). MicroRNA turnover: when, how and why. Trends Biochem. Sci. 37, 436–446. 10.1016/j.tibs.2012.07.002 22921610

[B49] Schluepmann H. Paul M. (2009). Trehalose metabolites in Arabidopsis – elusive, active and central, in The Arabidopsis Book, eds Last R. Chang C. Jander G. Kliebenstein D. McClung R. Millar H. Torii K. Wagner D. (Washington, DC: American Society of Plant Biologists), 1–17. 10.1199/tab.0122 PMC3243345 22303248

[B50] Schmutz J. McClean P. E. Mamidi S. Wu G. A. Cannon S. B. Grimwood J.. (2014). A reference genome for common bean and genome-wide analysis of dual domestications. Nat. Genet. 46, 707–713. 10.1038/ng.3008 24908249 PMC7048698

[B51] Searle I. R. Men A. E. Laniya T. S. Buzas D. M. Iturbe-Ormaetxe I. Carroll B. J.. (2003). Long-distance signaling in nodulation directed by a CLAVATA-like receptor kinase. Science 299, 109–112. 10.1126/science.1077937 12411574

[B52] Streeter J. G. (1988). Inhibition of legume nodule formation and N2-fixation by nitrate. CRC Crit. Rev. Plant Sci. 7, 1–23. 10.1080/07352688809382257

[B53] Suárez R. Wong A. Ramírez M. Barraza A. Orozco Md C. Cevallos M. A.. (2008). Improvement of drought tolerance and grain yield in common bean by overexpressing trehalose-6-phosphate synthase in Rhizobia. Mol. Plant-Microbe Interact. 21, 958–966. 10.1094/MPMI-21-7-0958 18533836

[B54] Sugawara M. Cytryn E. J. Sadowsky M. J. (2010). Functional role of *Bradyrhizobium japonicum* trehalose biosynthesis and metabolism genes during physiological stress and nodulation. Appl. Environ. Microbiol. 76, 1071–1081. 10.1128/AEM.02483-09 20023090 PMC2820964

[B55] Thibivilliers S. Joshi T. Campbell K. B. Scheffler B. Xu D. Cooper B.. (2009). Generation of *Phaseolus vulgaris* ESTs and investigation of their regulation upon Uromyces appendiculatus infection. BMC Plant Biol. 9:46. 10.1186/1471-2229-9-46 19397807 PMC2684537

[B56] Thompson J. D. Gibson T. J. Plewniak F. Jeanmougin F. Higgins D. G. (1997). The CLUSTAL_X windows interface: flexible strategies for multiple sequence alignment aided by quality analysis tools. Nucleic Acids Res. 25, 4876–4882. 10.1093/nar/25.24.4876 9396791 PMC147148

[B57] Trainer M. A. Capstick D. Zachertowska A. Lam K. N. Clark S. R. Charles T. C. (2010). Identification and characterization of the intracellular poly-3-hydroxybutyrate depolymerase enzyme PhaZ of *Sinorhizobium meliloti*. BMC Microbiol. 10:92. 10.1186/1471-2180-10-92 20346169 PMC2867953

[B58] Valdés-López O. Arenas-Huertero C. Ramírez M. Girard L. Sánchez F. Vance C. P.. (2008). Essential role of MYB transcription factor: PvPHR1 and microRNA: PvmiR399 in phosphorus-deficiency signalling in common bean roots. Plant Cell Environ. 31, 1834–1843. 10.1111/j.1365-3040.2008.01883.x 18771575

[B59] Vandesteene L. López-Galvis L. Vanneste K. Feil R. Maere S. Lammens W.. (2012). Expansive evolution of the trehalose-6-phosphate phosphatase gene family in Arabidopsis. Plant Physiol. 160, 884–896. 10.1104/pp.112.201400 22855938 PMC3461562

[B60] Van Houtte H. Vandesteene L. López-Galvis L. Lemmens L. Kissel E. Carpentier S.. (2013). Overexpression of the trehalase gene AtTRE1 leads to increased drought stress tolerance in Arabidopsis and is involved in abscisic acid-induced stomatal closure. Plant Physiol. 161, 1158–1171. 10.1104/pp.112.211391 23341362 PMC3585587

[B61] Varkonyi-Gasic E. Lough R. H. Moss S. M. Wu R. Hellens R. P. (2012). Kiwifruit floral gene APETALA2 is alternatively spliced and accumulates in aberrant indeterminate flowers in the absence of miR172. Plant Mol. Biol. 78, 417–429. 10.1007/s11103-012-9877-2 22290408

[B62] Vauclare P. Bligny R. Gout E. De Meuron V. Widmer F. (2010). Metabolic and structural rearrangement during dark-induced autophagy in soybean (Glycine max L.) nodules: an electron microscopy and 31P and 13C nuclear magnetic resonance study. Planta 231, 1495–1504. 10.1007/s00425-010-1148-3 20358222

[B63] Vessey J. K. (1994). Measurement of nitrogenase activity in legume root nodules: in defence of the acetylene reduction assay. Plant Soil 158, 151–162. 10.1007/BF00009490

[B64] Vlasova A. Capella-Gutiérrez S. Rendón-Anaya M. Hernández-Oñate M. Minoche A. E. Erb I.. (2016). Genome and transcriptome analysis of the Mesoamerican common bean and the role of gene duplications in establishing tissue and temporal specialization of genes. Genome Biol. 17, 32. 10.1186/s13059-016-0883-6 26911872 PMC4766624

[B65] Vogel G. Aeschbacher R. A. Müller J. Boller T. Wiemken A. (1998). Trehalose-6-phosphate phosphatases from *Arabidopsis thaliana*: identification by functional complementation of the yeast tps2 mutant. Plant J. 13, 673–683. 10.1046/j.1365-313X.1998.00064.x 9681009

[B66] Vogel G. Fiehn O. Jean-Richard-dit-Bressel L. Boller T. Wiemken A, Aeschbacher, R. A.. (2001). Trehalose metabolism in Arabidopsis: occurrence of trehalose and molecular cloning and characterization of trehalose-6-phosphate synthase homologues. J. Exp. Bot. 52, 1817–1826. 11520870 10.1093/jexbot/52.362.1817

[B67] Voinnet O. (2009). Origin, biogenesis, and activity of plant microRNAs. Cell 136, 669–687. 10.1016/j.cell.2009.01.046 19239888

[B68] Wahl V. Ponnu J. Schlereth A. Arrivault S. Langenecker T. Franke A.. (2013). Regulation of flowering by trehalose-6-phosphate signaling in *Arabidopsis thaliana*. Science 339, 704–707. 10.1126/science.1230406 23393265

[B69] Wang Y. Wang L. Zou Y. Chen L. Cai Z. Zhang S.. (2014). Soybean miR172c targets the repressive AP2 transcription factor NNC1 to activate ENOD40 expression and regulate nodule initiation. Plant Cell 26, 4782–4801. 10.1105/tpc.114.131607 25549672 PMC4311200

[B70] Wilson R. A. Gibson R. P. Quispe C. F. Littlechild J. A. Talbot N. J. (2010). An NADPH-dependent genetic switch regulates plant infection by the rice blast fungus. Proc. Natl. Acad. Sci. U.S.A. 107, 21902–21907. 10.1073/pnas.1006839107 21115813 PMC3003025

[B71] Wilson R. A. Jenkinson J. M. Gibson R. P. Littlechild J. A. Wang Z. Y. Talbot N. J. (2007). Tps1 regulates the pentose phosphate pathway, nitrogen metabolism and fungal virulence. EMBO J. 26, 3673–3685. 10.1038/sj.emboj.7601795 17641690 PMC1949003

[B72] Wingler A. Delatte T. L. O'Hara L. E. Primavesi L. F. Jhurreea D. Paul M. J.. (2012). Trehalose 6-phosphate is required for the onset of leaf senescence associated with high carbon availability. Plant Physiol. 158, 1241–1251. 10.1104/pp.111.191908 22247267 PMC3291265

[B73] Wollmann H. Mica E. Todesco M. Long J. A. Weigel D. (2010). On reconciling the interactions between APETALA2, miR172 and AGAMOUS with the ABC model of flower development. Development 137, 3633–3642. 10.1242/dev.036673 20876650 PMC2964095

[B74] Zhu Q. H. Helliwell C. A. (2011). Regulation of flowering time and floral patterning by miR172. J. Exp. Bot. 62, 487–495. 10.1093/jxb/erq295 20952628

