# Supplementary figures and images for: The Class II Trehalose 6-phosphate Synthase Gene PvTPS9 Modulates Trehalose Metabolism in Phaseolus vulgaris Nodules
Source: Front Plant Sci. 2016 Nov 1;7:1589. doi: 10.3389/fpls.2016.01589 (PMC5088437; doi:10.3389/fpls.2016.01589)

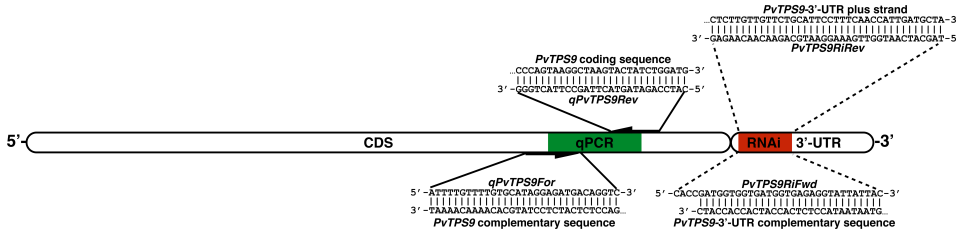

Supplement: Figure S1 — Schematic representation of the PvTPS9 cDNA sequence. The coding sequence (CDS) and the 3′untranslated region is shown in white boxes, and the DNA fragment amplified by qPCR and the target region to be silenced by RNAi are shown in green and red boxes, respectively. We also indicate the primer sequence and position to measure PvTPS9 transcript abundance by qPCR and to amplify the 300 bp DNA sequence used in our RNAi design. [file Image1.PDF]

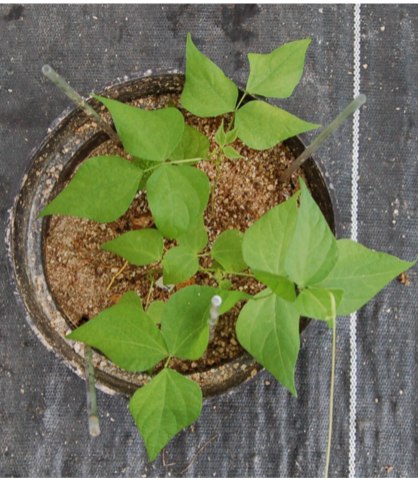

K599

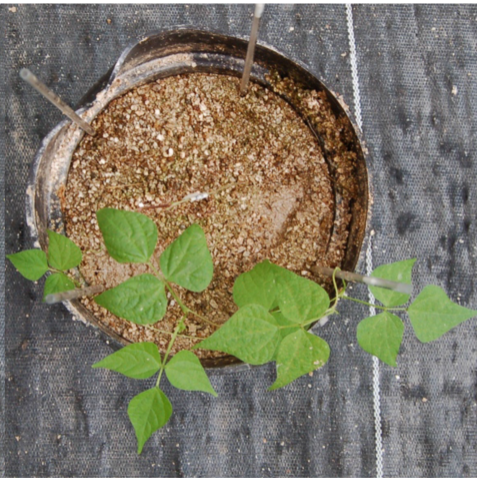

*P<sub>v</sub>TPS9-RNAi*

Supplement: Figure S2 — Control (A. rhizogenes K599) or PvTPS9-RNAi composite bean plants. Photograph was taken at 21 dpi. [file Image2.PDF]

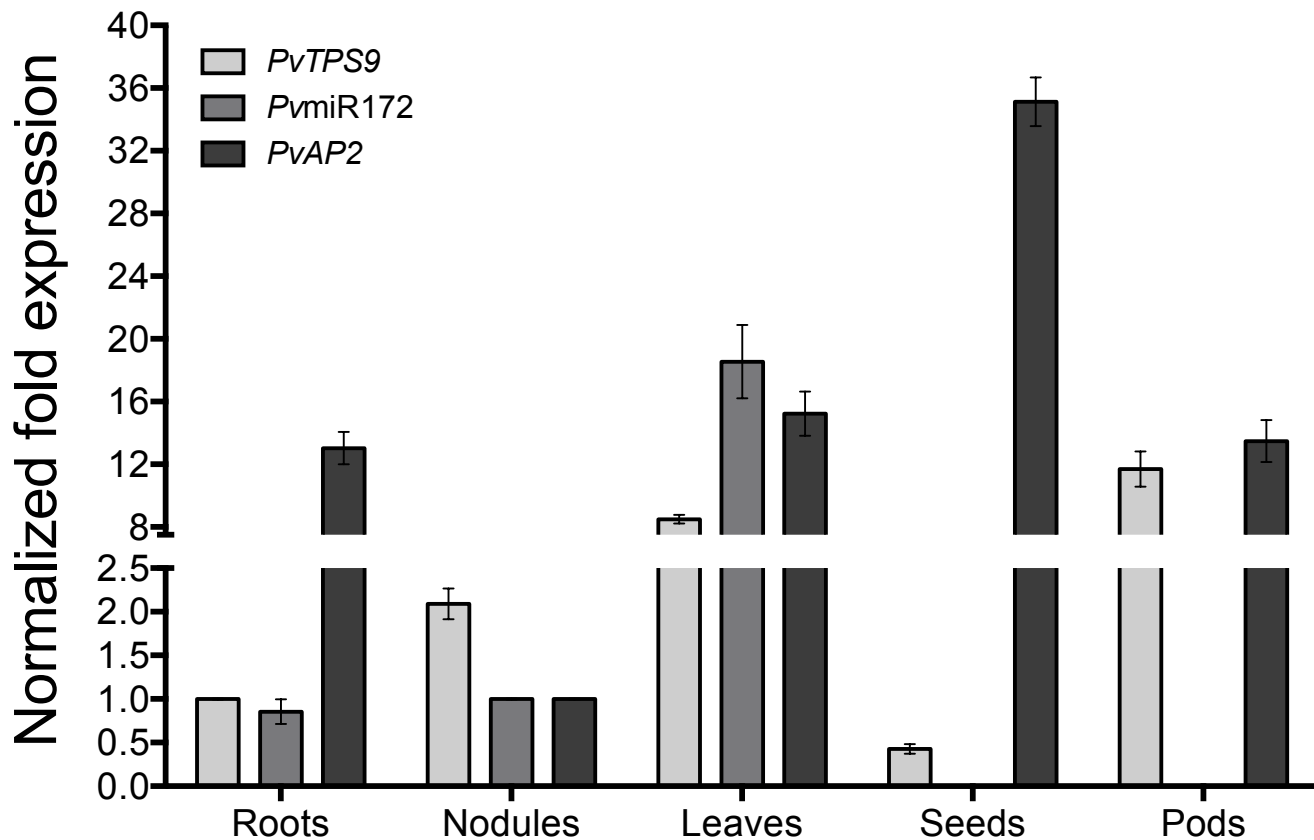

Supplement: Figure S3 — PvmiR172, PvAP2, and PvTPS9 transcript abundance determined in the different tissues of common bean plants by qPCR assays. Data came from plant tissues (roots, nodules, leaves, seeds and pods) of six independent (n = 6) biological replicates and shown as means ± SD. [file Image3.PDF]

(a)

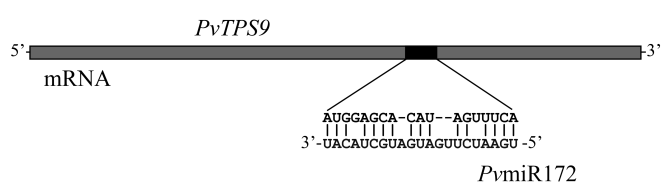

(b)

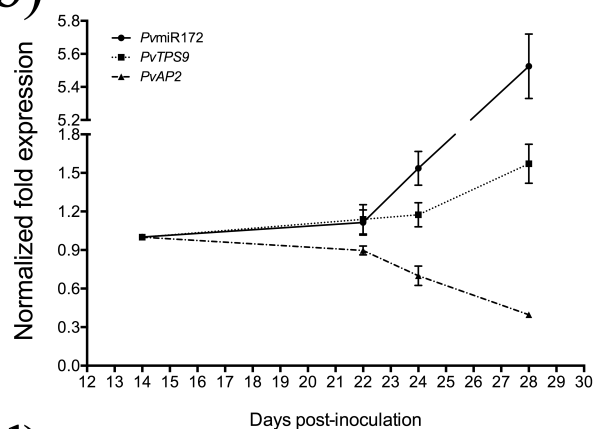

(c)

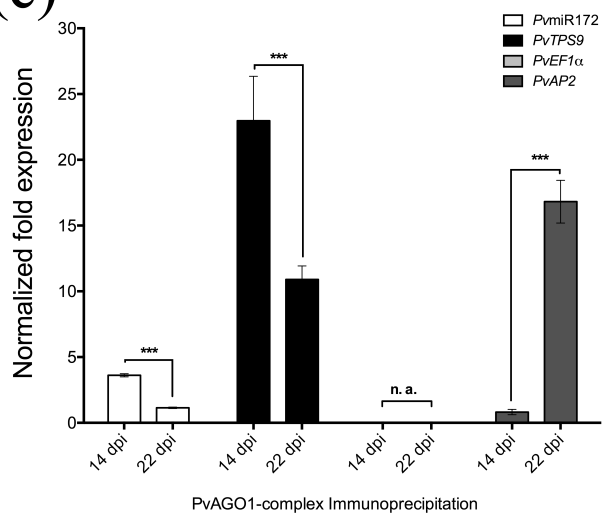

(d)

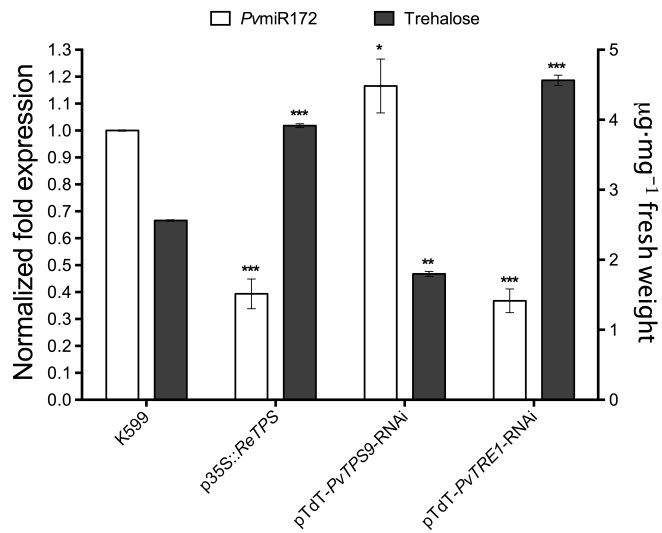

Supplement: Figure S4 — PvmiR172 expression could be regulated systemically by the trehalose content in P. vulgaris plants. (A) Schematic representation of the transcript sequence of PvTPS9 and the putative interaction with PvmiR172, showing three mismatches near the central zone of PvTPS9. (B) PvmiR172 (circles), PvAP2 (triangles), PvTPS9 (squares) gene expression profile during nodule development. qPCR data came from six independent (n = 6) wild-type nodules, tested by triplicate and normalized to the expression level of the Elongation factor 1-alpha (PvEF1a) gene. Plotted data are shown as mean ± SD. (C) Anti-PvAGO1 RNA co-immunoprecipitation (IP). Total extracts of 14 or 21 dpi wild-type nodules were divided in four parts and used for anti-PvAGO1 immunoprecipitation followed by RNA extraction and cDNA synthesis. qPCR data came from three (n = 3) independent experimental replicas tested by triplicate and normalized to the PvmiR2118 reference gene (Livak and Schmittgen, 2001). (D) PvmiR172 abundance and trehalose content in control (A. rhizogenes K599) or in p35S::ReTPS, PvTPS9-RNAi and PvTRE1-RNAi transgenic nodules of composite bean plants. qPCR data came from nodules of six independent (n = 6) transgenic roots. The statistical signficance was determined with an unpaired two-tailed Student's t-test (*P < 0.05, **P < 0.01, ***P < 0.001), and shown as means ± SD. [file Image4.PDF]

(a)

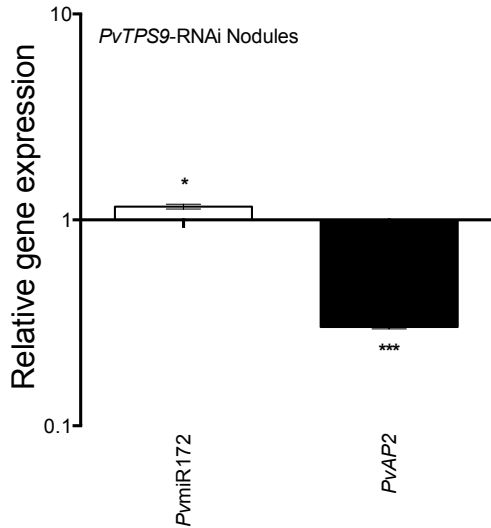

(b)

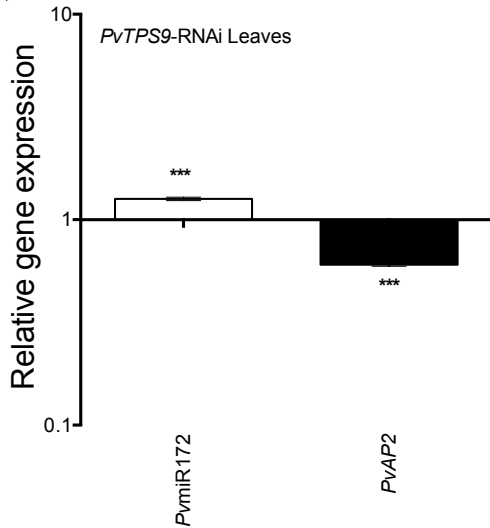

Supplement: Figure S5 — PvmiR172 and PvAP2 transcript abundance in leaves of PvTPS9-RNAi composite common bean plants by qPCR assays. (A,B) Statistical significance was determined with an unpaired two-tailed Student's t-test (*P < 0.05; ***P < 0.001). Plotted data are expressed as log10 of relative gene expression of Class II TPS genes in the control composite common bean plants and shown as means ± SD. [file Image5.PDF]
