# Supplementary material for: The Class II Trehalose 6-phosphate Synthase Gene PvTPS9 Modulates Trehalose Metabolism in Phaseolus vulgaris Nodules
Source: Front Plant Sci. 2016 Nov 1;7:1589. doi: 10.3389/fpls.2016.01589 (PMC5088437; doi:10.3389/fpls.2016.01589)
Supplement: Table S1 — List of primers used in this work. [file Table1.DOCX]

| **Primer Name** | **Primer Sequence (5’ to 3’)** | **Target** |
| --- | --- | --- |
|  |  |  |
| q*Pv*EF1aFor | GGTCATTGGTCATGTCGACTCTGG | *Pv*EF1a |
| q*Pv*EF1aRev | GCACCCAGGCATACTTGAATGACC |  |
|  |  |  |
| qGUSFor | TAACCACGCGTCTGTTGACTG | β-Glucuronidase |
| qGUSRev | GGTTGCCAGAGGTGCGGATTCA |  |
|  |  |  |
| q*Pv*TPS9For | ATTTTGTTTTGTGCATAGGAGATGACAGGTC | *Pv*TPS9 |
| q*Pv*TPS9Rev | CATCCAGATAGTACTTAGCCTTACTGGG |  |
|  |  |  |
| q*Pv*GOGATFor | ATGTCTTCATCAATCTCGTTTCCCACG | *Pv*GOGAT (NADH Glutamate Synthase II) |
| q*Pv*GOGATRev | ACGCAACCGTGTTCCCAAAAACTTTGTC |  |
|  |  |  |
| q*Pv*GSFor | AGCACCAAGACAATGAGAAATGATG | *Pv*GS (Glutamine Synthetase) |
| q*Pv*GSRev | GTCGTCCTGTCAGACGGCG |  |
|  |  |  |
| q*Pv*AP2For | ATT CTC AAT TTC AGC TAC CTT TC | *Pv*AP2 (Apetala2) |
| q*Pv*AP2Rev | TGA CCT CGT AAT AGA ATT GAG G |  |
|  |  |  |
| q*Pv*miR172For | TGAATCTTGATGATGCTGCAT | *Pv*miR172 |
|  |  |  |
| q*Pv*miR156For | TGACAGAAGAGAGAGAGCACA | *Pv*miR156 |
|  |  |  |
| q*Pv*TPS4For | CACTTCAGAGTTACGAGCGAATTGCTT | *Pv*TPS4 |
| q*Pv*TPS4Rev | GGTCTGTCCCTAAGATCTCATCTATC |  |
|  |  |  |
| q*Pv*TPS5For | CTGAAACTTCTAGCCGTGGAACATC | *Pv*TPS5 |
| q*Pv*TPS5Rev | CATTCTTTGCTTCTTGAACATCCTTCCCA |  |
|  |  |  |
| q*Pv*TPS6For | CCCACGAGCAGGAAGTAGTTGCT | *Pv*TPS6 |
| q*Pv*TPS6Rev | GAAAGAGAGGCCAAAGCTGCTGTT |  |
|  |  |  |
| q*Pv*TPS7For | CCTTCAGCTCAAAGACGGGTTCCCTT | *Pv*TPS7 |
| q*Pv*TPS7Rev | CGTTGGGACGCAGCGAAACCTTTC |  |
|  |  |  |
| q*Pv*TPS8For | CTAATAGGGATAAAACTTTACACATTGGTTC | *Pv*TPS8 |
| q*Pv*TPS8Rev | GGCAAAAGCATTAATATCCCATGGGTT |  |
|  |  |  |
| q*Pv*TPS10For | CAGAAACATAAGCCTGCCACTGTGATCTAT | *Pv*TPS10 |
| q*Pv*TPS10Rev | CTTGCCTACTTTTATTCCTCCAGAACTCTACA |  |
|  |  |  |
| *Pv*TPS9RiFwd | CACCGATGGTGGTGATGGTGAGAGGTATTATTAC | *Pv*TPS9-3'-UTR for RNAi |
| *Pv*TPS9RiRev | TAGCATCAATGGTTGAAAGGAATGCAGAACAAGAG |  |
|  |  |  |
| WRKY-5-Rev | GCAGAGGAGGAGAAGCTTCTAG | WRKY-Intron pTdT-DC-RNAi derived constructs to confirm fragment orientation |
|  |  |  |
|  |  |  |
| *Re*TPSFor | CCATGGGCCGTCTTATCGTCGTTTCCAATCG | *Rhizobium etli* TPS |
| *Re*TPSRev | AGATCTGATCCGTGCGCCCCGCTCCCATC |  |
