# Supplementary material for: The Class II Trehalose 6-phosphate Synthase Gene PvTPS9 Modulates Trehalose Metabolism in Phaseolus vulgaris Nodules
Source: Front Plant Sci. 2016 Nov 1;7:1589. doi: 10.3389/fpls.2016.01589 (PMC5088437; doi:10.3389/fpls.2016.01589)
Supplement: Table S2 — Class I, Class II, and Class III gene families involved in the biosynthesis of trehalose in common bean (Phytozome v11, http://www.phytozome.net; Mazorka, http://mazorka.langebio.cinvestav.mx/blast/). [file Table2.DOCX]

| **Protein classification** | **Gene identifier** | **Gene name** |
| --- | --- | --- |
| Class I | Phvul.011G170300.1 | *PvTPS1* |
|  | Phvul.005G076500.2 | *PvTPS2* |
|  | Phvul.002G243600.1 | *PvTPS3* |
| Class II | Phvul.007G031800.1 | *PvTPS4* |
|  | Phvul.003G053000.1 | *PvTPS5* |
|  | Phvul.002G072400.1 | *PvTPS6* |
|  | Phvul.003G150400.1 | *PvTPS7* |
|  | Phvul.003G198800.1 | *PvTPS8* |
|  | Phvul.003G016300.1 | *PvTPS9* |
|  | Phvul.009G180300.1 | *PvTPS10* |
| Class III | Phvul.011G006000.1 | *PvTPPA* |
|  | Phvul.003G218200.1 | *PvTPPB* |
|  | Phvul.005G008300.1 | *PvTPPC* |
|  | Phvul.001G251300.1 | *PvTPPD* |
|  | Phvul.002G254600.1 | *PvTPPE* |
|  | Phvul.008G172400.1 | *PvTPPF* |
|  | Phvul.009G054400.1 | *PvTPPG* |
|  | Phvul.010G112900.1 | *PvTPPH* |
|  | Phvul.009G029800.1 | *PvTPPI* |
|  |  |  |
